# Nanotechnology-augmented sonodynamic therapy and associated immune-mediated effects for the treatment of pancreatic ductal adenocarcinoma

**DOI:** 10.1007/s00432-022-04418-y

**Published:** 2022-11-02

**Authors:** Marym Mohammad Hadi, Sian Farrell, Heather Nesbitt, Keith Thomas, Ilona Kubajewska, Alex Ng, Hamzah Masood, Shiv Patel, Fabiola Sciscione, Brian Davidson, John F. Callan, Alexander J. MacRobert, Anthony P. McHale, Nikolitsa Nomikou

**Affiliations:** 1 Division of Surgery and Interventional Science, Faculty of Medical Sciences, University College London, London, UK; 2 Biomedical Sciences Research Institute, Ulster University, Coleraine, UK; 3 Nanomerics Ltd, London, UK

**Keywords:** Sonodynamic therapy, Pancreatic cancer, Nanoparticles, Hematoporphyrin, Cathepsin B, Anticancer immune response

## Abstract

**Purpose:**

Sonodynamic therapy (SDT) is emerging as a cancer treatment alternative with significant advantages over conventional therapies, including its minimally invasive and site-specific nature, its radical antitumour efficacy with minimal side effects, and its capacity to raise an antitumour immune response. The study explores the efficacy of SDT in combination with nanotechnology against pancreatic ductal adenocarcinoma.

**Methods:**

A nanoparticulate formulation (HPNP) based on a cathepsin B-degradable glutamate-tyrosine co-polymer that carries hematoporphyrin was used in this study for the SDT-based treatment of PDAC. Cathepsin B levels in BxPC-3 and PANC-1 cells were correlated to cellular uptake of HPNP. The HPNP efficiency to induce a sonodynamic effect at varying ultrasound parameters, and at different oxygenation and pH conditions, was investigated. The biodistribution, tumour accumulation profile, and antitumour efficacy of HPNP in SDT were examined in immunocompetent mice carrying bilateral ectopic murine pancreatic tumours. The immune response profile of excised tumour tissues was also examined.

**Results:**

The HPNP formulation significantly improved cellular uptake of hematoporphyrin for both BxPC-3 and PANC-1 cells, while increase of cellular uptake was positively correlated in PANC-1 cells. There was a clear SDT-induced cytotoxicity at the ultrasound conditions tested, and the treatment impaired the capacity of both BxPC-3 and PANC-1 cells to form colonies. The overall acoustic energy and pulse length, rather than the power density, were key in eliciting the effects observed in vitro. The SDT treatment in combination with HPNP resulted in 21% and 27% reduction of the target and off-target tumour volumes, respectively, within 24 h. A single SDT treatment elicited an antitumour effect that was characterized by an SDT-induced decrease in immunosuppressive T cell phenotypes.

**Conclusion:**

SDT has significant potential to serve as a monotherapy or adjunctive treatment for inoperable or borderline resectable PDAC.

**Supplementary Information:**

The online version contains supplementary material available at 10.1007/s00432-022-04418-y.

## Background

Sonodynamic therapy (SDT) has emerged as a promising approach for the minimally invasive treatment of recalcitrant solid organ cancers, particularly those associated with poor-prognosis and resistance to conventional therapies such as chemotherapy and radiotherapy. SDT involves the application of low-intensity ultrasound that generates cytotoxic reactive oxygen species (ROS), such as singlet oxygen and other radical species, in the presence of sensitizing agents, via the chemical interaction with molecular oxygen and/or biological and other molecules (McHale et al. 2016; Hadi et al. 2021). The cytotoxicity of intracellular ROS has been mainly attributed to mitochondrial damage and lipid peroxidation that can induce cell death via apoptosis or necrosis.

Based on its attributes, SDT is suggested as an attractive alternative to chemotherapy and radiotherapy, for the treatment of inoperable or borderline resectable pancreatic ductal adenocarcinoma (PDAC). The low-intensity ultrasound beam required for the treatment can target a pancreatic lesion using either endoscopic or extracorporeal transducers, through appropriate tissue acoustic window(s) (Göbl et al. 2017). In SDT, ultrasound is transmitted efficiently through tissue and can irradiate large tumours more effectively than X-ray based radiotherapy, with minimal collateral damage to the surrounding healthy tissues. In addition, SDT has exhibited good efficacy even under hypoxic conditions, in which radiotherapy has exhibited impaired efficacy (Hadi et al. 2021). Unlike in SDT, the issues of systemic toxicity, multidrug resistance and heterogeneity of PDAC soon render chemotherapy inefficient in neoadjuvant treatment, while both radiotherapy and chemotherapy are associated with extensive immunosuppression that can exacerbate disease progression. Interestingly, an increasing number of studies have focussed on the induction of an anti-tumour immune response following SDT, a phenomenon that has been studied using immunocompetent mice carrying syngeneic tumours (Peng et al. 2018; Nicholas et al. 2021). Moreover, some studies have demonstrated enhanced SDT efficacy using immune-checkpoint inhibitors such as anti-PD1/PD-L1 and anti-CTLA-4 (Yue et al. 2019; Nesbitt et al. 2021).

The sensitizing molecules most commonly used in preclinical and clinical SDT studies are water-soluble derivatives of porphyrin (e.g. HPD) or chlorin (chlorin e6), which are administered systemically and reach the target tumour mass via blood circulation. It is well-established that nanotechnology can improve the accumulation profile of sensitizing molecules in cancerous tissue (Nomikou et al. 2017). The benefits afforded to the active agent include and are not limited to increased blood circulation time, improved cellular uptake, favourable subcellular localization profile, and, most importantly, the improved tumour accumulation via the enhanced permeability and retention effect, which is unique to cancerous tissues. The characteristics of the tumour microenvironment play a major role in the delivery of therapeutic agents/nanoparticles, and consequently, the efficacy of certain treatment modalities for different cancers. PDAC is one of the most recalcitrant forms of cancer that renders conventional treatments, such as chemotherapy and radiotherapy, inefficient mainly due to the low vascularization, and hypoxic and immunosuppressive nature of the PDAC microenvironment (Tao et al. 2021). Smart nanotechnology systems are designed to respond to tumour-specific stimuli, such as the acidic pH and/or the elevated concentrations of proteolytic enzymes, to afford site-specific agent release within the tumour mass and/or improved cellular uptake (Thomas et al. 2020). For the afore-mentioned reasons, the majority of recent SDT studies employ sensitizer-carrying multifunctional nanoparticulate systems that have shown exceptionally better performance in facilitating an improved sonodynamic effect, compared to the free sensitizers.

Hadi et al. described the development of a nanoparticulate system (HPNP) carrying hematoporphyrin (HP), formed through the self-assembly of the sensitizer with a glutamate-tyrosine co-polymer (poly(l-glutamic acid-l-tyrosine) 4:1) (Hadi et al. 2021). It was demonstrated that the HPNP formulation responds to a number of characteristics of the tumour microenvironment, providing enhanced activity of the sensitizer in response to ultrasound over that provided by the free agent. It was shown that digestion of the nanoparticles with cathepsin B, an enzyme that is overexpressed and secreted in the tumour microenvironment, leads to decreased nanoparticle size and subsequent increased cellular uptake. The formulation was used efficiently for the sonodynamic treatment of prostate cancer target cell populations, under both normoxic and hypoxic conditions. In vivo studies using immunodeficient mice, demonstrated that SDT using the HPNP decreased LNCaP tumour volumes by 64% within 24 h, following intravenous administration of a single HPNP dose. No adverse effects were recorded, and body weight was stable.

In the current study, the efficacy of SDT using the HPNP nanoparticulate platform was investigated for use in SDT-based treatment of pancreatic cancer. For in vitro studies, the human PDAC cell lines BxPC-3 and PANC-1 were used, due to their respective epithelial and mesenchymal properties. In vivo experiments were performed using immunocompetent mice bearing ectopic bilateral pancreatic tumours with the objective of studying the impact of treatment on target and distant, off-target tumours, as well as the treatment-induced anticancer immune response.

## Materials and methods

### Cell culture

BxPC-3, a human pancreatic cancer cell line, and T110299, a cell line isolated from primary pancreatic tumours in KPC mice (Ptfla-Cre; *LSL*-*Kras*^G12D^; *LSL-Trp*^53fl/R172H^), were a gift from Prof. Jens Siveke (Klinikum rechts der Isar, Technical University Munich, Germany). BxPC-3 cells were maintained in RPMI Medium 1640 (1x) (Life Technologies, UK) supplemented with L-Glutamine (GibcoBRL,UK), 10% v/v foetal bovine serum (FBS) (Life Technologies, UK) and 1% penicillin/streptomycin (Merck, UK), in a humidified 5% CO_2_/20% O_2_ atmosphere, at 37 °C. The T110299 murine cell line was maintained in DMEM medium containing high glucose (4.5 g/L) (Life Technologies, UK), supplemented with 10% (v/v) FBS, 1% (v/v) penicillin/streptomycin and 1% (v/v) non-essential amino acids (Merck, UK) solution in a humidified 5% CO_2_/20% O_2_ atmosphere, at 37 °C. PANC-1, a human pancreatic cell line isolated from pancreatic carcinoma of ductal cell origin, was obtained from the American Type Culture Collection (ATCC). This cell line was maintained in DMEM medium containing high glucose (4.5 g/L) (Life Technologies, UK), supplemented with 10% (v/v) FBS and 1% (v/v) penicillin/streptomycin in a humidified 5% CO_2_/20% O_2_ atmosphere, at 37 °C. When required, single-cell suspensions were prepared by treating cell monolayers with a 0.05% w/v solution of trypsin containing 0.02% w/v EDTA in phosphate-buffered saline (PBS). Incubation at pH 6.4 was performed in growth medium containing 0.15% v/v of lactic acid 85% w/v solution. The in vitro cytotoxicity of the treatments was defined on the basis of cell viability, which was determined indirectly by measuring the relative metabolic activity using an MTT assay, 24 h after SDT treatment, as described by Hadi et al. (2021).

### Measuring intracellular and extracellular cathepsin B activity

BxPC-3, PANC-1, and T110299 cells were seeded in 24-well plates at a concentration of 6 × 10^4^ cells per well and incubated for 24 h, in a humidified 5% CO_2_/20% O_2_ atmosphere, at 37 °C. The growth medium was then removed, and the systems incubated at either normoxic (20% O_2_) conditions and pH 7.4 or at hypoxic (1% O_2_) conditions and pH 6.4, for 24 h. A fluorometric cathepsin B activity assay kit (Abcam) was used to determine intracellular and extracellular cathepsin B activity from lysed cells and the corresponding growth medium recovered from the culture wells, respectively. The growth medium was recovered for extracellular cathepsin B analysis. For intracellular activity, cells were lysed using cell lysis buffer, (Abcam) based on the manufacturer’s instructions. The lysate and extracellular medium samples were both normalised based on total protein concentration (mg/mL), measured using a NanoDrop OneC UV–Vis spectrophotometer (ThermoFisher Scientific). Subsequently in the wells of a 96-well plate, 50 µL of lysate/extracellular medium sample, 50 μL of cathepsin B reaction buffer and 2 µL of cathepsin B substrate were added. The plates were then incubated for 45 min, and fluorescence was measured using an Infinite M200 Pro Multimode Microplate Reader (Tecan, Switzerland) at 400 nm and 505 nm excitation and emission wavelengths, respectively (Ex/Em = 400/505 nm).

### Cellular uptake

The HPNP formulation was manufactured as described by Hadi et al. (2021), via the self-assembly of HP with poly(l-glutamic acid-l-tyrosine) 4:1, sodium salt (pGATyr) (Merck, UK). To study the cellular uptake of free HP and HPNP, cells were seeded in 96-well plates at a density of 2 × 10^4^ per well and incubated in a humidified 5% CO_2_/20% O_2_ atmosphere, at 37 °C, for 24 h. Cells were then incubated with growth medium (FBS-supplemented) containing each HP formulation (free HP or HPNP), either at normoxic conditions and pH 7.4 or at hypoxic conditions and pH 6.4, for 24 h. The HP (free and nanoparticulate) concentrations used in these systems were 10 μg/mL for BxPC-3 cells and 15 μg/mL for PANC-1 cells, across all conditions. These HPNP concentrations were the maximum concentrations of HPNP with a relatively low toxicity for each cell line (Fig. S1) and were determined with a “silent” toxicity assay (Supplementary material, Sect. 1.1). The cell monolayers were then washed twice with PBS, 100 μL DMSO was added, and the systems were incubated for 45 min, followed by pipette-mixing. The samples were examined using fluorescence spectrophotometry, at 534 nm and 626 nm excitation and emission wavelengths, respectively. The fluorescence emission values were normalised against the control wells (in the absence of free HP or HPNP) and on the basis of the cell viability in correspondingly treated separate cultures, determined using the MTT assay.

### Invasion assay

The membrane of the inserts in 24-Transwell plates (Costar) were pre-coated with Matrigel (Corning, UK), using 40 μL of the latter diluted to 0.125 μg/mL with cold sterile dH_2_O. Prior to seeding 2 × 10^4^ cells per insert, BxPC-3 and PANC-1 cells were starved from serum for 24 h. Then, 100 μL HPNP in serum-free growth medium (SFM) was added in the upper chamber at a final concentration of 10 μg/mL for BxPC-3 and 15 μg/mL for PANC-1. The pGATyr concentration in SFM (100 μL) in the upper chamber corresponded to the polymer content of the HPNP amount that was added for each cell line (99.9 μg/mL for BxPC-3 and 133.2 μg/mL for PANC-1). Control systems contained SFM only. 600 μL of complete RPMI or DMEM medium was added in the lower chamber. The systems were then incubated for 48 h, at 37 °C, in a 5% CO_2_/20% O_2_ humidified incubator. The excess cells that had not invaded through the membrane of the insert were removed with cotton swab. Subsequently, the inserts were fixed with neat methanol and stained with crystal violet (Fluka BioChemika, UK). The number of invaded cells were manually counted using a microscope.

### In vitro SDT treatment

BxPC-3, PANC-1 and T110299 cells were seeded in 96-well plates at a concentration of 2 × 10^4^ cells per well and incubated for 24 h in a humidified 5% CO_2_/20% O_2_ atmosphere, at 37 °C. Growth medium was then replaced with medium (FBS-supplemented) containing the HPNP formulation, at 10 μg/mL for BxPC-3, 15 μg/mL for PANC-1 and 15 μg/mL for T110299. Cell systems were incubated at either normoxic conditions and pH 7.4, normoxic conditions and pH 6.4 or hypoxic conditions and pH 6.4, for 24 h. The treatment medium was then removed, the cell monolayers were washed with PBS and fresh growth medium was added. Target cells were exposed to ultrasound (US) at a frequency of 1 MHz, at a pulse repetition frequency of 100 Hz, at varying power density (intensity) and with varying duty cycle (DC, %) for 30 s using an SP100 sonoporator (Sonidel Ltd. Ireland). The treatment parameters correspond to the settings of the device. For US exposure/treatment, the transducer of the SP100 was placed in direct contact with the bottom of the wells using contact gel. Control systems consisted of untreated, US only-treated and HPNP only-treated target cells. The systems were then incubated for 24 h under their respective normoxic or hypoxic conditions. Cell viability was determined using the MTT assay. The sonodynamic effect was calculated using the formula:${\text{SDT effect}}\, = \,\% {\text{SDT toxicity}}{-}(\% {\text{HPNP toxicity}}\, + \,\% {\text{US toxicity}}).$

### Clonogenic assay

BxPC-3 and PANC-1 cells were seeded and treated with SDT, at 3 W/cm^2^ and 50% DC, for 30 s, as described in the previous Section. Incubations were at normoxic conditions and pH 7.4. 24 h post treatment, in order to examine the formation of colonies, a single cell suspension was generated from each system using trypsinization. Each suspension was washed with medium and cells from each group were then transferred into individual wells of 6-well plates at the cell seeding density of 1350 cells per well (number defined on the basis of the cell suspension with the lowest cell number among all test systems) with 3 ml of growth medium. The systems were then incubated at the same conditions as before, for 7–14 days, until the control system (no treatment) formed colonies with at least 50 cells. After incubation, the cells in colonies were fixed with 1 mL of 4% paraformaldehyde (Merck, UK) for 10 min and then stained with 5% w/v crystal violet for 15 min. Following staining, the colonies were washed with distilled water and were left to dry overnight before being manually counted.

### Biodistribution and tumour accumulation kinetics

All animals employed in this study were treated in accordance with the licensed procedures under the UK Animals (Scientific Procedures) Act 1986. T110299 cells were subcutaneously implanted in the right and left rear dorsum of 6–8 week old C57BL/6 mice. Each injection for implantation consisted of 5 × 10^5^ cells in 100 μL PBS. Tumour volume was measured with Vernier callipers and was calculated using the formula (width × length × height)/2. When tumours reached an average volume of 150 mm^3^, animals were anaesthetized using intraperitoneal administration of Hypnorm/Hypnovel and 100 μL of HPNP suspension were administered intravenously by tail vein injection, at a dose of 1.2 mg/mL in PBS (approx. 6 mg/Kg). Following administration, animals were placed in the chamber of a Xenogen IVIS® Lumina imaging system on fluorescence mode using the DsRed filter set (excitation: 500–550 nm; emission: 575–650 nm). The data for each region of interest (ROI) outlined in the images captured were analysed using the Living Image^®^ software package version 2.60 and presented as arbitrary fluorescence units using the fluorescence efficiency mode to eliminate illumination intensity effects. For ex vivo imaging to examine the tissue distribution of nanoparticles, animals were sacrificed at different time points after HPNP administration and organs were surgically excised. Organs were then placed in the imaging system and fluorescence data were captured in a similar manner.

### In vivo sonodynamic treatment

Bilateral subcutaneous T110299 tumours on the right and left rear dorsum of C57BL/6 mice were induced as described in the previous Section. When tumours had reached an average size of 150 mm^3^, animals were randomly distributed into groups for treatment with HPNP alone, US alone and HPNP plus US. Control animals that received no treatment were also employed as a separate group. Animals were anaesthetized during all treatments, using intraperitoneal administration of 1:2 dilutions of a 1:1 mixture of Hypnorm/Hypnovel. The animals treated with HPNP received 100 μL aliquots of the suspension at a dose of 1.2 mg/mL in PBS via intravenous administration. Animals were then rested for 24 h and then tumours on the right (target tumours) were exposed to US at a frequency of 1 MHz, for 3.5 min, a pulse repetition frequency of 100 Hz, 3.5 W/cm^2^ (SATP) and 50% DC, for 3.5 min. The volumes of both target and off-target (on the left) tumours were monitored at the indicated times as described in the previous Section.

### Immune response analysis

Animals treated as described in the previous section were sacrificed on days 4 and 11, residual tumour tissues were excised and converted into single cell suspensions in RPMI supplemented with 4% FBS, 160 μL (30 mg/mL) collagenase type II, 50 μL (2 μg/mL) DNAse, during incubation at room temperature, in a high-speed shaker, for 15 min. The mixture was filtered through a 100 μm filter, centrifuged at 17,000 rpm for 5 min and the supernatant was decanted. Residual red blood cells in the samples were removed using a multi-species RBC lysis buffer, as per manufacturer's instructions. Cells were then washed twice in ice cold PBS and were stained with eBioscience fixable viability dye (eFluor 520) (for determining viability), fluorochrome conjugated antibodies specific for CD45 (PE-Cy7/0.125 μg/test), CD3 (APC-eFluro 780/1 μg/test), CD4 (Alexa Fluor 700/0.125 μg/test), CD8a (PE/0.25 μg/test), CD25 (PE-Cy5/0.125 μg/test) and CTLA4 (PE-eFluor610/0.5 μg/test), in PBS containing 0.2% (w/v) bovine serum albumin for 30 min, at room temperature. Subsequently samples were fixed and permeabilised by eBioscience FoxP3 staining buffer set, as per manufacturer’s instructions, and stained with fluorochrome conjugated antibody specific for FoxP3 (APC/1 μg/test), in PBS containing 0.2% (w/v) bovine serum albumin for 30 min at room temperature. Viable cells were determined as eBioscience fixable viability dye negative and single cells were identified by plotting FS-H versus FS-A. Cytotoxic T cells were identified as CD45 + CD3 + CD8a + cells. Helper T cells were identified as CD45 + CD3 + CD4 + cells. Data generated were analysed using FlowJo (BD Biosciences), with fold-change in treatment groups calculated in comparison to the untreated ones. The gating strategy is summarised in Supplementary Table S1. All antibodies and reagents were sourced from ThermoFisher (UK).

### Statistical analysis

All statistical analysis was carried out using GraphPad prism (versions 5 and 8.4.2) for Windows. Data were expressed as means ± standard error of the mean (SEM). Column data were analysed using One-way ANOVA with the Tukey multiple comparison test. Grouped data were analysed using a Two-way ANOVA with a Bonferroni post-test. * denotes *p* < 0.05, ** denotes *p* < 0.01, *** denotes *p* < 0.001, ns denotes no significance.

For the immune response analysis data, the differences between means of two groups were compared by two-tailed unpaired Student’s *t* test or Mann–Whitney *U* test (if the data distribution was non-normal). The effect size was calculated for statistically significant differences as a quantitative measure of the magnitude of a phenomenon, complementary to statistical hypothesis testing. Cohen’s *d* was computed as the measure of effect size. Glass's Delta and Hedges' *G* were included for reference as alternative measures. By convention, the magnitude of an effect was interpreted as “small” when Cohen’s *d* was between 0.2 and 0.49; “medium” effect for *d* = 0.5–0.79; and “large” effect for *d* ≥ 0.8, according to benchmarks suggested originally by Cohen (Lakens 2013).

## Results

### Cathepsin B assay, cellular uptake of the hematoporphyrin-carrying nanoparticles and the effect of the latter on cell invasion

**Fig. 1:**
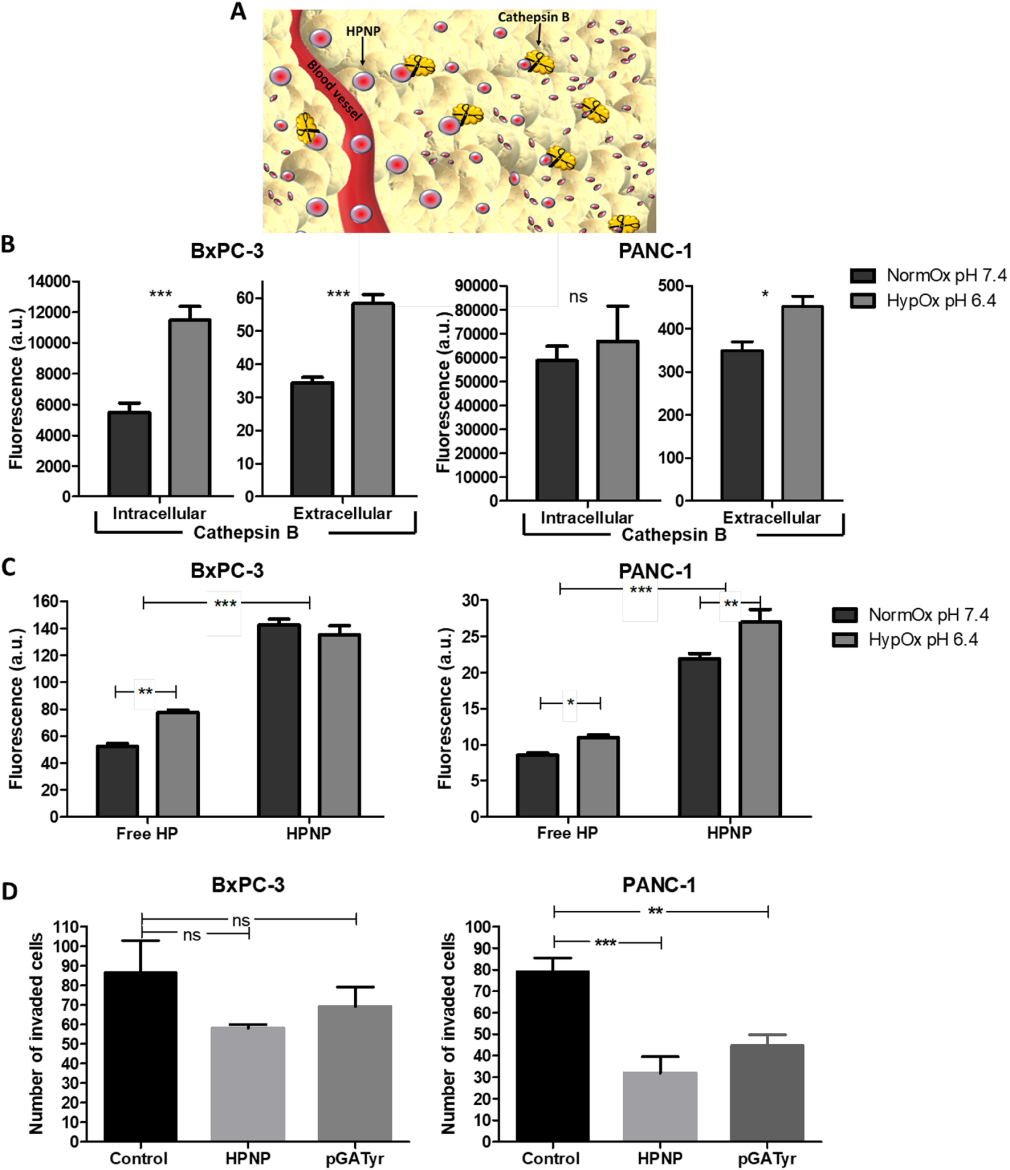
**A** Nanoparticle digestion by cathepsin B upon extravasation within a tumour. **B** Levels of extracellular and intracellular cathepsin B after 24 h incubation of BxPC-3 and PANC-1 cells at normoxic conditions and pH 7.4 (NormOx 7.4), and at hypoxic conditions and pH 6.4 (HypOx 6.4) (*n* = 4). **C** Cellular uptake after incubation of BxPC-3 and PANC-1 cells with free hematoporphyrin (free HP) and nanoparticles (HPNP) for 24 h, at the corresponding conditions (*n* = 4). **D** Invasion assay for BxPC-3 and PANC-1 cells treated with the nanoparticles (HPNP) and pGATyr (*n* = 3). Statistical significance was computed using *t* test (**B**), Two-way ANOVA with Bonferroni post-test (**C**), and One-way ANOVA with Tukey multiple comparison test (**D**) (*ns *not significant, *p < 0.05, **p < 0.01 and ***p < 0.001). Error bars represent ± the SD

Clinical evidence has indicated that PDAC has a considerably dense fibrotic stroma that hampers the perfusion of systemically administered agents throughout the tumour mass (Tao et al. 2021). Experimental measurements and mathematical models have suggested that nano-carriers with smaller sizes can afford higher accumulation in the tumour tissue interstitium. Their small size allows them to penetrate through to non-vascularized regions, so they can expose a larger portion of the tumour to the payload (Soltani et al. 2021). The physicochemical characteristics of the HPNP have been previously explored, and their mean diameter was measured to be 55 nm (Hadi et al. 2021). It was demonstrated that the size of the HPNP decreased in the presence of cathepsin B, a proteolytic enzyme overexpressed in the tumour microenvironment (Fig. 1A), while in vitro cellular uptake was correlated with the production and secretion of cathepsin B.

Here, the production and secretion of cathepsin B by the human pancreatic cell lines, BxPC-3 and PANC-1 cells, at physiological and acidic pH, were determined and the correlation of the findings with cellular uptake was investigated. Data in Fig. 1B demonstrate that, for BxPC-3 cells, the levels of intracellular and extracellular cathepsin B were significantly increased (110% and 69% increase, respectively) at hypoxic and acidic conditions, when compared with normoxic conditions at physiological pH. However, the increased levels of cathepsin B had no effect on the cellular uptake of the HPNP (*p* > 0.05) (Fig. 1C). This result is not in agreement with previous findings using a prostate cancer cell line, where increased levels of cathepsin B were proportionally correlated to cellular uptake of HPNP (Hadi et al. 2021). However, it should be noted that the level of extracellular cathepsin B for BxPC-3 cells at acidic conditions was particularly low, when compared to those of PANC-1 and T110299 cells (Fig. 1C, Supplementary Fig. S2, respectively). For PANC-1 cells, both intracellular and extracellular cathepsin B levels increased (14% and 29% increase, respectively) at hypoxic and acidic conditions, when compared with normoxic conditions at physiological pH, and in this case, cellular uptake of HPNP was also increased by 26%. For both BxPC-3 and PANC-1 cells, cellular uptake of hematoporphyrin was significantly improved when the sensitizer was incorporated in the HPNP formulation (*p* < 0.001) (Fig. 1C).

The cell invasion study (Fig. 1D) demonstrated that both the HPNP formulation and the polymeric nanoparticle component, pGATyr that served as HP carrier, significantly impaired cell invasion capacity for PANC-1 cells by 52% on average, at non-toxic concentrations (Supplementary Fig. S3). A similar trend was observed in the case of BxPC-3 cells, although this was not found to be statistically significant (*p* = 0.0611).

### In vitro sonodynamic effect

**Fig. 2:**
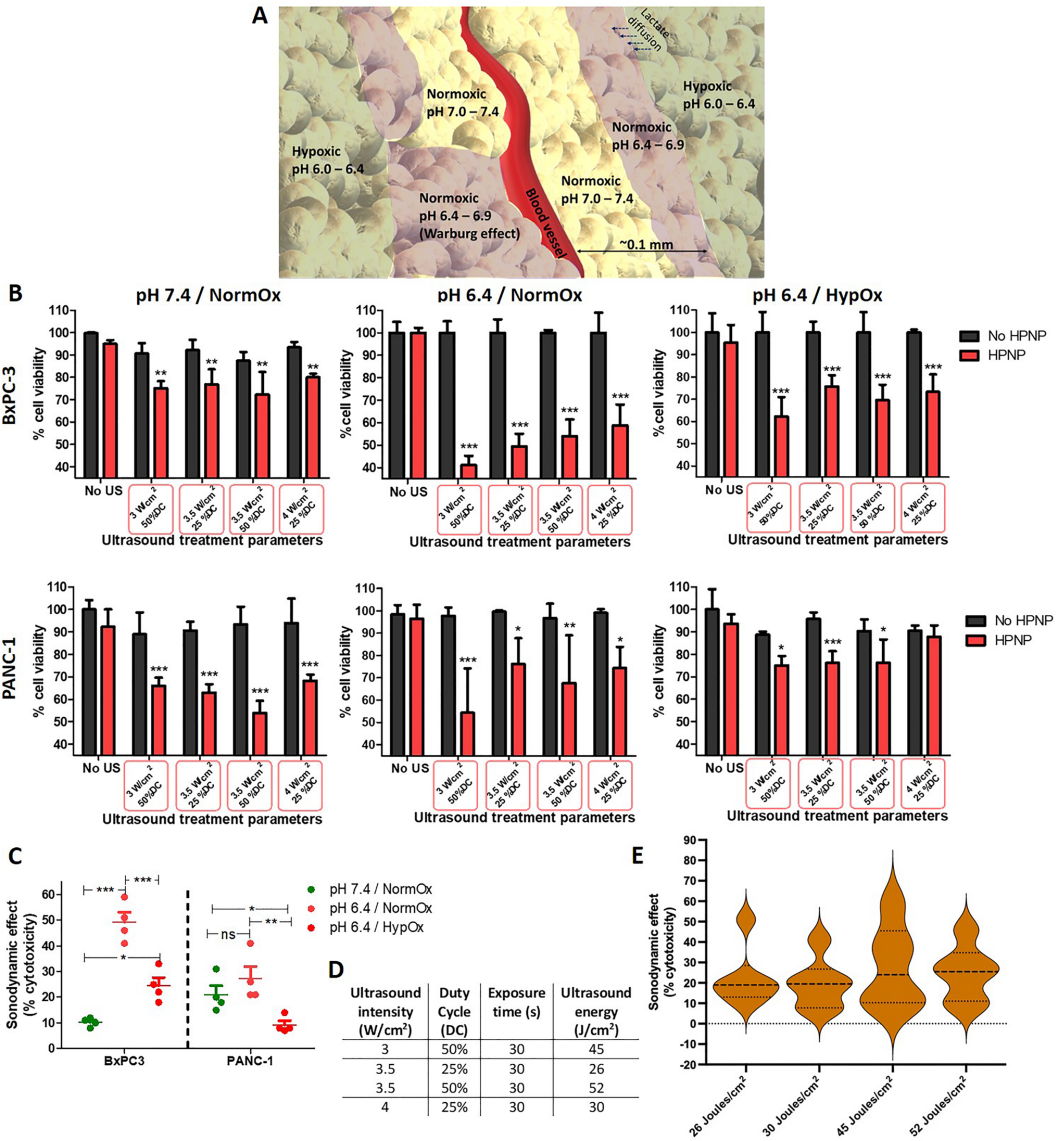
Sonodynamic treatment of BxPC-3 and PANC-1 cells. **A** pH gradient around a tumour blood vessel. **B** % cell viability of BxPC-3 and PANC-1 cells treated in the absence (no HPNP) and the presence of nanoparticles (HPNP), without (no US) and with ultrasound exposure (30 s) at different parameters, at pH 7.4 and normoxic (pH 7.4/NormOx), pH 6.4 and normoxic (pH 6.4/NormOx) and pH 6.4 and hypoxic (pH 6.4/HypOx) conditions. **C** The impact of pH and oxygenation conditions on the effect of SDT in % cytotoxicity, independently from ultrasound exposure parameters. **D** Conversion of ultrasound intensity applied in the systems per unit area to acoustic energy per unit area. **E** Violin plot depicting the impact of the applied ultrasound energy (Joules/cm^2^) on the effect of SDT in % cytotoxicity, independently from the cell type, pH and oxygenation conditions. Statistical significance was computed using Two-way ANOVA with Bonferroni post-test (**B**) and One-way ANOVA with Tukey multiple comparison test (**C**) (*ns *not significant, *p < 0.05, **p < 0.01, ***p < 0.001). For **B**: the asterisks show the significance of difference between samples incubated in the presence and the absence of nanoparticles, under ultrasound exposure. Error bars represent ± the SD, where *n* = 4

During exposure of a target tumour to US, the acoustic energy delivered to different regions within a pancreatic tumour mass is expected to vary due to varying US attenuation caused by tumour heterogeneity that is linked to the uneven density (collagen concentration), the existence of necrotic regions, the presence of inflammation and the irregular vascularization (Civale et al. 2006). US attenuation within tissue leads to the decrease of both wave intensity and energy density at a fixed time, and consequently is expected to influence the sonodynamic effect against target tumour cells. Although US exposure parameters used in vitro cannot be fairly translated to an in vivo “closed” system, it is important to investigate how the US treatment parameters and the potential attenuation through tissue may affect the efficacy of SDT using human pancreatic cancer target cell lines. The SDT efficacy also depends on the level of target tissue oxygenation, considering that a large proportion of the SDT-induced ROS formation depends on the presence of molecular oxygen. Pancreatic adenocarcinoma tumours are dominated by hypoxic regions, in which pH is particularly acidic due to glucose metabolism through glycolysis and the subsequent accumulation of lactate in the extracellular space (Fig. 2A). Increased lactic acid production by tumour cells has also been detected in well-oxygenated tumour regions, and it is due to a process called “aerobic glycolysis” or Warburg effect (Guillaumond et al. 2013). In addition, the lactate produced in hypoxic regions can diffuse towards better oxygenated ones, forming a pH gradient from the normoxic to the hypoxic areas. Therefore, studying the efficacy of SDT using the HPNP at varying US conditions and varying pH/oxygenation (environmental conditions) is important for determining the potential of the treatment to affect a pancreatic tumour throughout its mass. To that end, BxPC-3 and PANC-1 cells were treated with the HPNP at the hematoporphyrin-based concentrations of 10 μg/mL and 15 μg/mL (Fig. S1), respectively, at three different cell culture conditions: normoxic/pH 7.4, normoxic/pH 6.4, and hypoxic/pH 6.4. The treatment was combined with US exposure 24 h later, at combinations of parameters with negligible cell damage (cytotoxicity) in the absence of HPNP and a positive sonodynamic effect in the presence of the HPNP.

The results demonstrate that, overall, there was a clear SDT-induced cytotoxicity at the US conditions tested for both target cell lines (Fig. 2B). Increasing the US intensity did not improve the treatment-induced cytotoxicity and the intensity of 4 W/cm^2^ yielded the lowest effect on cell viability. The sonodynamic effect is defined as the synergistic cytotoxic effect between US and nanoparticles, when combined for the treatment of a target cell population. Both cell lines showed improved response to SDT at normoxic and pH 6.4 conditions, where the cumulative sonodynamic effect, independently from the US parameters tested, was 49% for BxPC-3 cells and 27% for PANC-1 cells (Fig. 2C). The results on the cumulative sonodynamic effect of the acoustic energies applied, independently from the environmental conditions, showed that the treatment outcome for both cell lines was improved when higher total acoustic energy (Joules/cm^2^) was applied to the targets. More specifically, US at 3 W/cm^2^ at 50% duty cycle (DC) and 3.5 W/cm^2^ at 50% DC, that yielded the highest energies at 45 J/cm^2^ and 52 J/cm^2^, respectively (Fig. 2D), facilitated improved sonodynamic effect, compared to the other two sets of parameters applied (Fig. 2E). These data suggested that within the acoustic power density range examined in these studies, the total acoustic energy adjusted by the pulse length, rather than the acoustic power, was key in eliciting the observed effects.

**Fig. 3:**
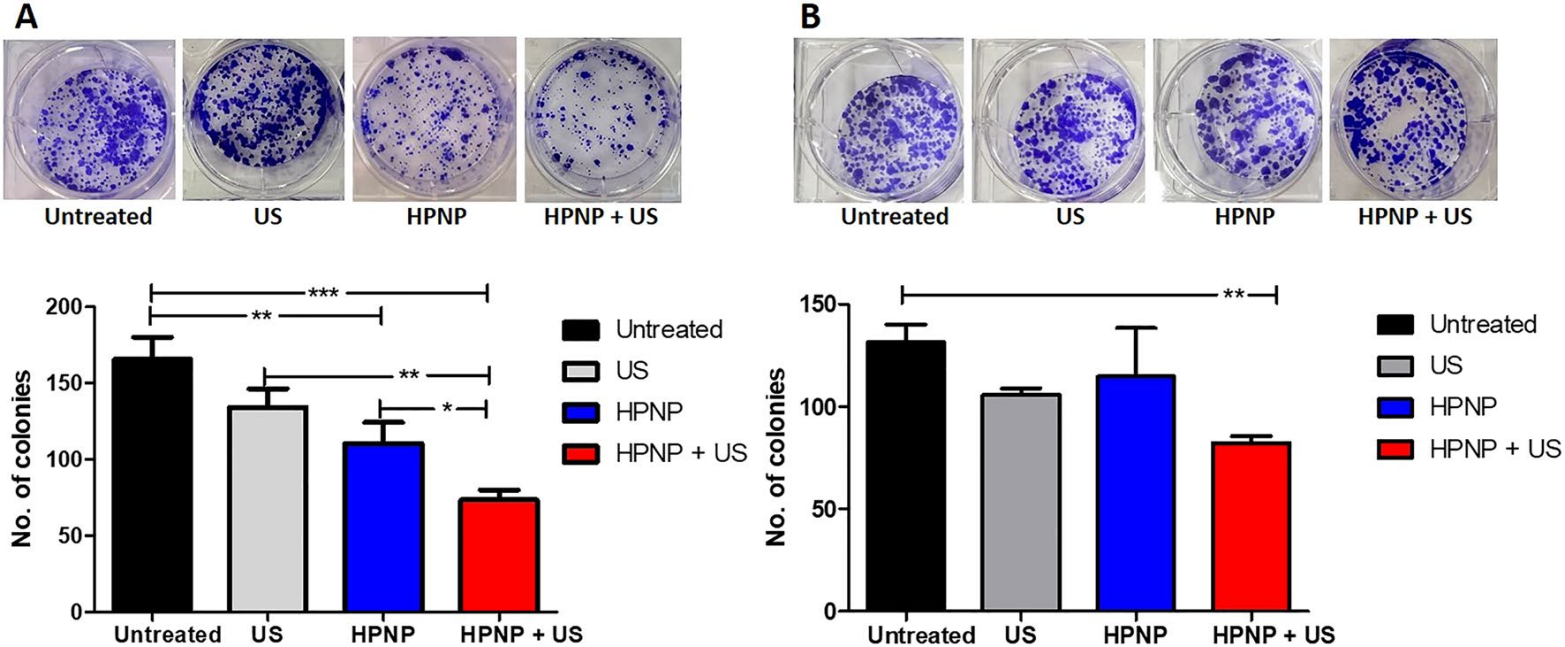
Clonogenic assay for BxPC-3 and PANC-1 cells treated with SDT. The number of colonies from untreated, ultrasound only-treated (US), nanoparticle only-treated (HPNP) and SDT-treated BxPC-3 (**A**) and PANC-1 (**B**) cells, with the corresponding representative images of colonies. Statistical significance was computed using Two-way ANOVA with Bonferroni post-test (*p < 0.05, **p < 0.01, ***p < 0.001). Error bars represent ± the SD, where *n* = 4

The effect of SDT using the HPNP formulation on the capacity of BxPC-3 and PANC-1 cells to form colonies was examined using a clonogenic assay. Data in Fig. 3 demonstrate that SDT decreased the ability of treatment-surviving cells to form colonies for both cell lines. More specifically, 44% of BxPC-3 cells that survived treatment with SDT had the ability to generate colonies and this percentage was increased to 61% for PANC-1 cells. These results are in line with the data obtained with the MTT assay in Fig. 2B where the PANC-1 seemed to be more resistant to SDT.

### In vivo SDT efficacy studies

**Fig. 4:**
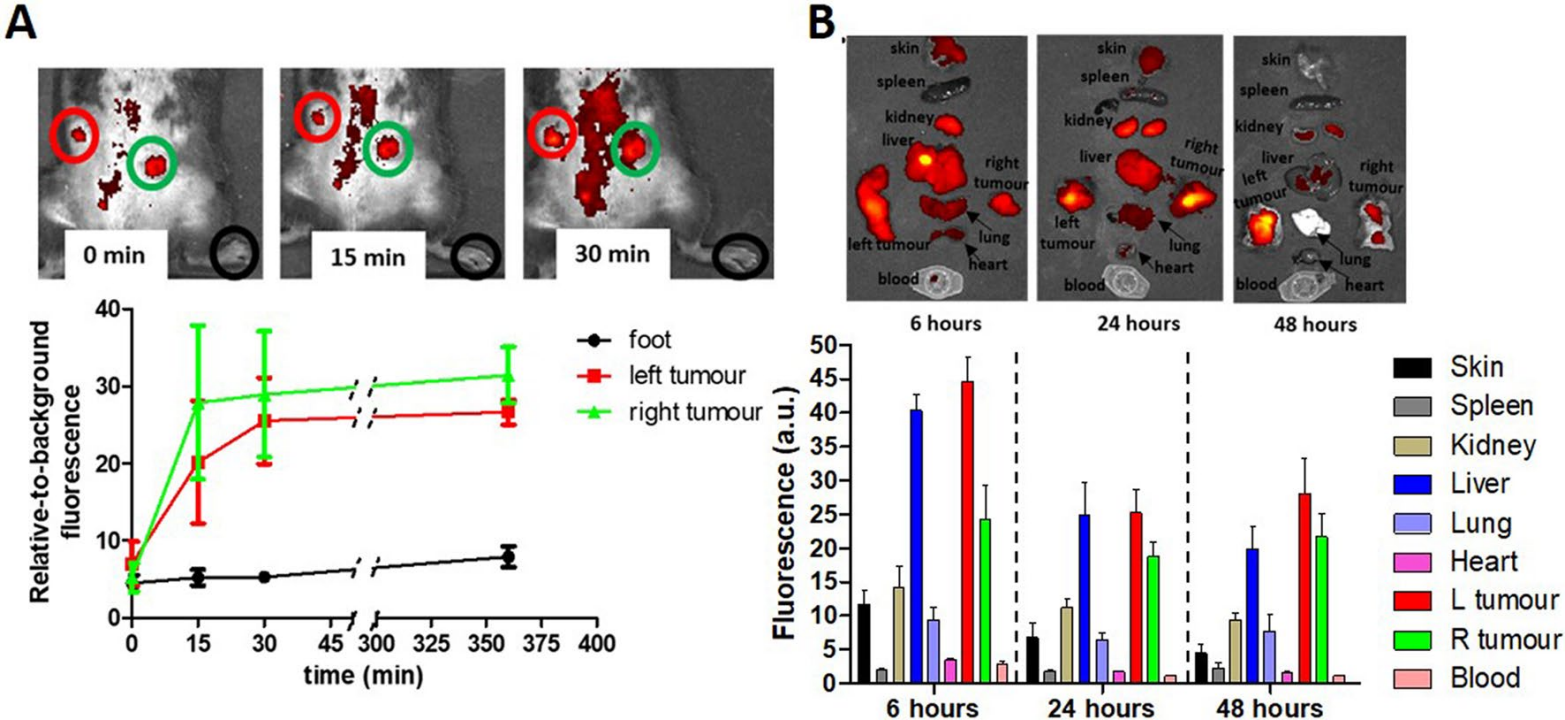
Biodistribution studies for the nanoparticles injected in C57BL/6 mice bearing bilateral T110299 tumours. **A** Fluorescence imaging signal from tumours and peripheral tissues (foot), normalised with the background signal (relative-to-background fluorescence), at different time points after nanoparticle injection. The inserts are corresponding representative images for different time points showing the nanoparticle distribution throughout the body in a single animal. ROIs for the left and right tumour are circled in red and green, respectively, and ROIs for the foot are circled in black, **B** Fluorescence imaging of surgically harvested tissues and representative images at different time points. Error bars represent ± the SD, where *n* = 5

One of the major advantages of SDT in the treatment of cancer is its non-hyperthermic ablative nature, which results in the release of intact tumour-associated antigens that can generate an effective anti-tumour immune response. The latter can significantly contribute to the overall effect of SDT in eliminating cancer and could potentially be exploited in treating metastatic disease. In order to examine the potential of a HPNP-mediated, SDT-induced anti-tumour immune response, in vivo experiments were performed using immunocompetent mice bearing ectopic bilateral pancreatic tumours. The tumour cell line employed to generate the tumour model was derived from KPC mice (Niknafs et al. 2019). It carries both an activating mutation in *Kras* and a dominant negative mutation in *Trp53*, commonly found in human PDAC. Such models established from KPC mice-derived cells retain important features of human PDAC tumours, such as desmoplasia, low vascularity and an immunosuppressive microenvironment (Pham et al. 2021). Prior to proceeding with the sonodynamic treatment of the tumour-bearing mice, it was important to establish the biodistribution and tumour accumulation profile of the HPNP upon systemic administration. Whole-body fluorescence imaging was performed, and a strong signal, suggesting rapid HP uptake, was immediately evident in both tumours (Fig. 4A). The fluorescence signal from peripheral tissues was also detected as noted from the hind leg at different time points and this was used as a surrogate for the widespread circulatory distribution of the HPNP. As shown in Fig. 4A, signals detected from tumour masses and the peripheral tissues, normalized on the basis of background signal (relative-to-background fluorescence), demonstrated a rapid increase in fluorescence in both tumours within the first 30 min after injection, and this was sustained for the following 6 h. Over this time period, it was noted that the signal from the peripheral tissues remained weak. In order to confirm that the HPNP were being accumulated in the tumour and to establish their tissue distribution, the excised organs and tumour tissues harvested from animals that were euthanised at varying time points after HPNP administration were also imaged to determine the relative nanoparticle load indicated by the fluorescence signal. Data in Fig. 4B demonstrate that at all time points, the left tumour emitted the highest fluorescence signal on average, among all organs and tissues at all time points. The signal from the right tumour exceeded that of all organs, apart from the liver at 24 h, while its average signal was higher than the signal from the liver at 48 h. Although these data are semi-quantitative, they clearly demonstrate the improved tumour accumulation profile of the HPNP formulation when compared to other nanoparticulate platforms described in the literature for the delivery of agents to similar tumour model (Das et al. 2019).

**Fig. 5:**
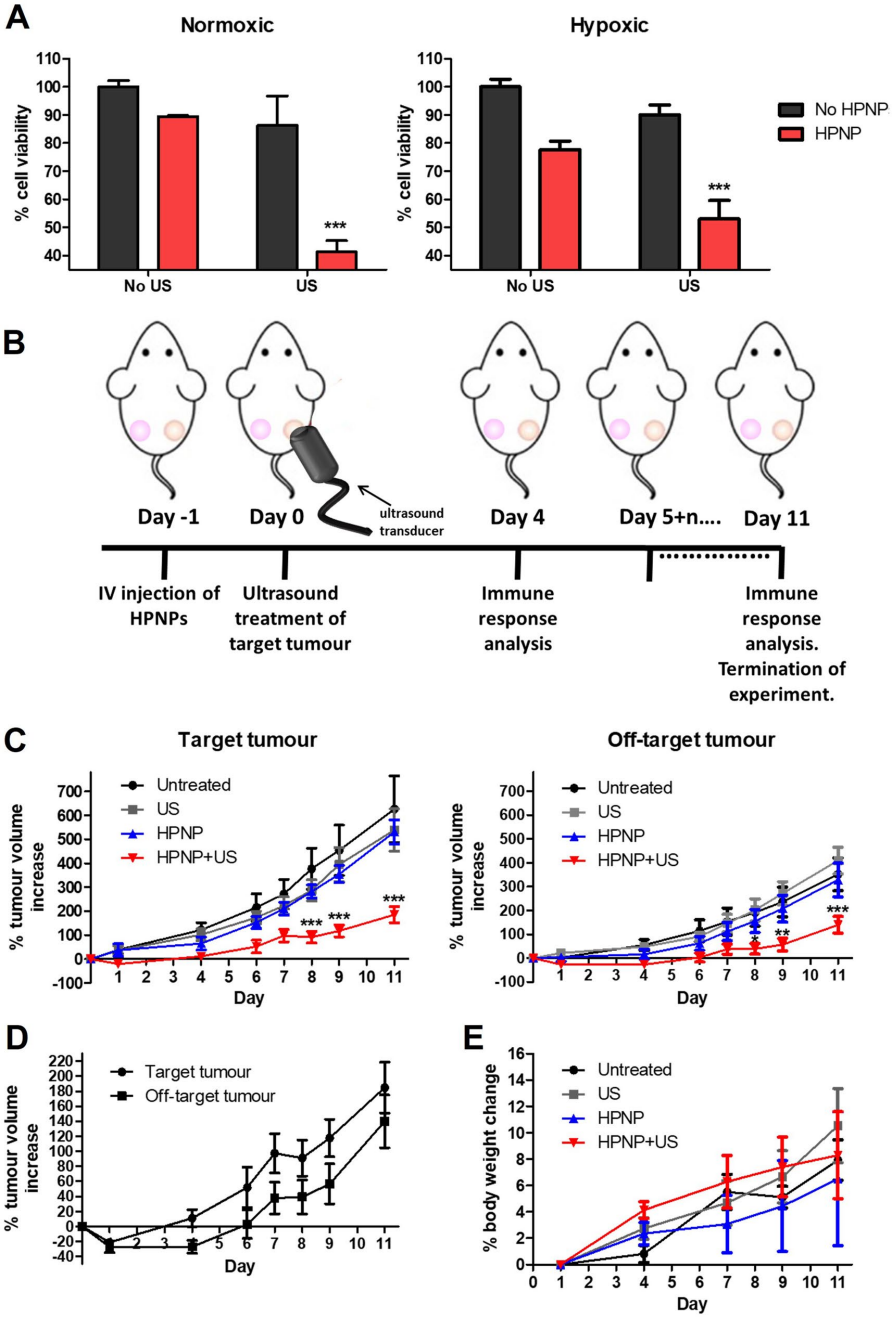
Sonodynamic treatment of T110299 tumours. **A** % cell viability of T110299 cells treated in the absence (no HPNP) and the presence of nanoparticles (HPNP), without (no US) and with ultrasound exposure at 3 W/cm^2^ and 50% DC, for 30 s, at normoxic and hypoxic conditions. **B** In vivo treatment protocol. **C** Plot of % change of target and off-target tumour volumes treated with no treatment (untreated), ultrasound only (US), nanoparticles carrying hematoporphyrin (HPNP) and nanoparticles carrying hematoporphyrin with ultrasound, i.e. sonodynamic therapy, SDT, (HPNPs + US). **D** Plot of % change of target and off-target tumour volumes trated with SDT. **E** The corresponding animal body weight increase. Statistical significance was computed using Two-way ANOVA with Bonferroni post-test (**A**) and One-way ANOVA with Tukey multiple comparison test (**C**) (*p < 0.05, **p < 0.01, ***p < 0.001). For **A**: the asterisks show the significance of difference between samples incubated in the presence and the absence of nanoparticles, under ultrasound exposure. Error bars represent ± the SD, where *n* = 5

Although the sonodynamic performance of the HPNP platform against BxPC-3 and PANC-1 cells was established in vitro earlier in this study, it was essential to confirm that the sensitizing formulation also supports the sonodynamic treatment of T110299 cells forming the target tumour, and that this type of cells responds to the treatment. Subsequently, further in vitro experimentation was carried out with T110299 as the target cell line and data in Fig. 5A confirm that this cell type responds to SDT in a similar manner as the human pancreatic cancer cell lines tested in this study. More specifically, SDT, US at 3 W/cm^2^ and 50% DC, decreased cell viability by 47% and 59% at hypoxic and normoxic conditions respectively, at pH 6.4.

Subsequent in vivo SDT efficacy studies were performed, where the HPNP formulation was administered intravenously to the bilateral tumour-bearing animals at a dose of 6 mg/kg. Approximately 24 h after IV administration of the nanoparticles, target tumours (right-hand side tumours) were treated with US (Fig. 5B). In this study, the US exposure dose employed in previous studies using this and similar nanoparticle formulations was applied (Hadi et al. 2021; Nomikou et al. 2017). The data obtained are shown in Fig. 5C (graphs with the raw volume data are provided in Supplementary Fig. S4) and they clearly demonstrate that, while administration of the HPNP or US alone had no impact on growth of either tumour (target or off-target), treatment using the HPNP formulation in combination with US resulted in 21% and 27% reduction in growth of the target and off-target tumour volume, respectively, within 24 h. Importantly, the SDT-treated target and off-target tumours reached their initial pre-treatment size on day 3 and day 6, respectively, (where the corresponding growth curves cross the baseline), when in control animals, the tumour size had grown beyond the 91% and 115% of the initial size. Interestingly, the effect of SDT on the mean size reduction was higher for the off-target tumour (27%) compared to the target tumour (21%) on day 1, while the growth profile of the target and off-target tumours were similar (Fig. 5D). In addition, the body weight profiles of the animals treated with the HPNP formulation only or with SDT were similar to the untreated and US-only control groups and no obvious adverse effects were observed during experimentation, which serves to illustrate the safety of both the systemically administered formulation and the combination treatment with US (Fig. 5E).

### Immune mechanisms of SDT antitumour activity

**Fig. 6:**
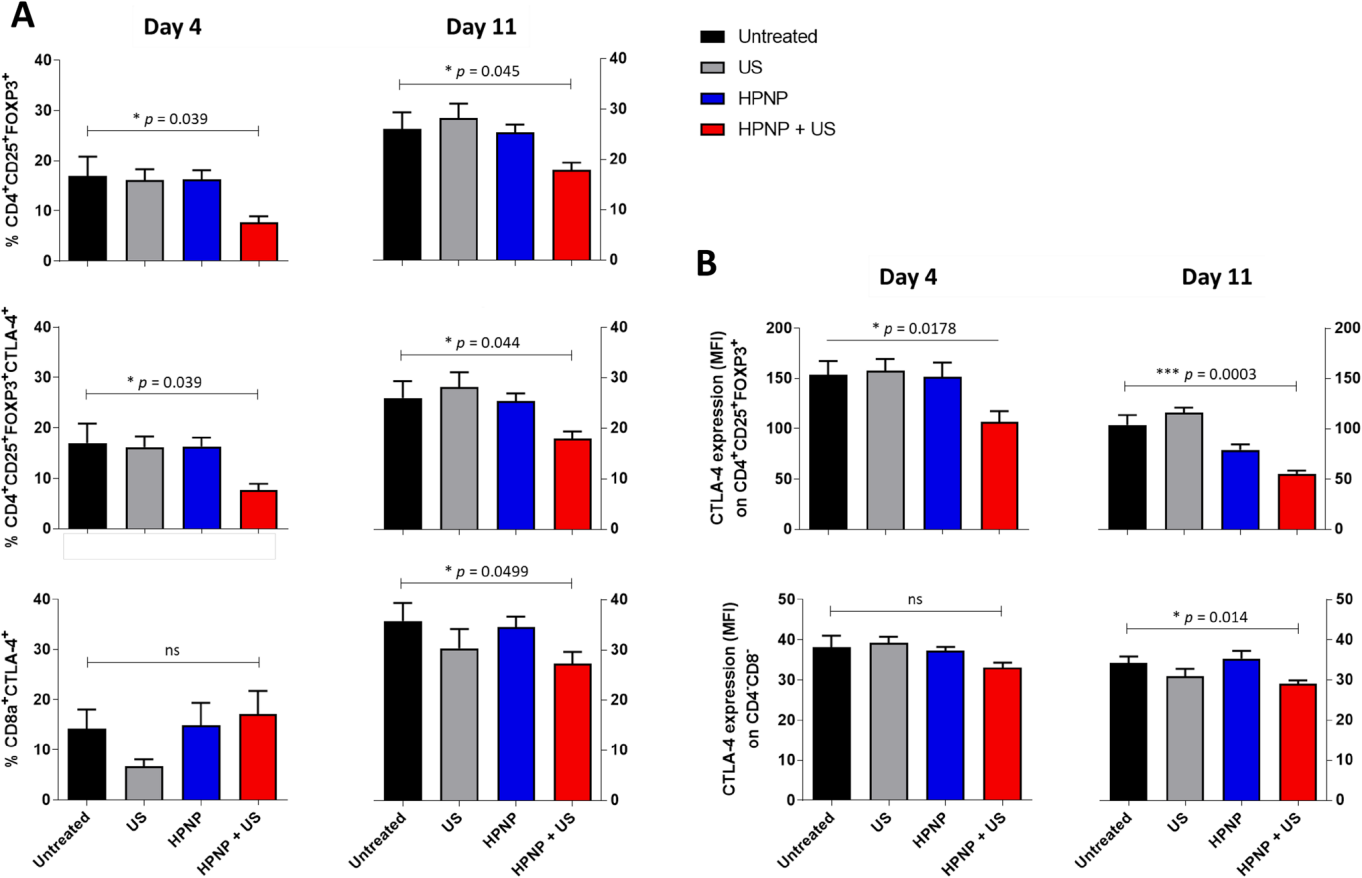
Inhibition of regulatory T cells subsets (**A**) and their CTLA-4 expression (**B**) in total tumour tissue in C57BL/6 mice in response to the SDT treatment (day 4 versus day 11). Tumours were collected from mice on days 4 and 11 and cells were isolated and phenotyped by FACS analysis. Bar graphs show the percentages of indicated cell phenotypes within CD45 ^+^ CD3 ^+^ T cell population (**A**) or CTLA-4 expression on given cell subsets based on mean fluorescence intensity (MFI) (**B**). Statistical significance was computed using Student’s *t* test or Mann–Whitney *U* test (*ns *not significant, *p < 0.05, **p < 0.01 and ***p < 0.001). Data are expressed as mean ± SEM (*n* = 8)

Subsequent analysis in the present study sought to characterize the immune cell phenotypic profile that infiltrated the target and off-target tumours as a result of the SDT treatment described here. To understand the underlying mechanism of the antitumour effects triggered by SDT using the HPNP formulation, immune cells from the tumour tissues were analysed on days 4 and 11 after treatment. The analysis was specifically focused on regulatory T cells (Tregs) and their expression of immune checkpoint cytotoxic T-lymphocyte associated protein 4 (CTLA-4) as key contributors to tumour immune evasion, which undermines antitumour immune responses (Facciabene et al. 2012; Weber 2008). A range of T cell subsets exhibiting suppressor phenotypes within CD45^+^CD3^+^ population, including CD4^+^, CD8^+^ and double negative CD4^−^CD8^−^ Tregs, was analysed. It was observed that the SDT-mediated depletion of total tumour volume coincided with changes in the proportions of different tumour-infiltrating Treg subsets and their CTLA-4 expression (as depicted in Fig. 6A, B, respectively). Firstly, high percentages of CD45^+^CD3^+^ T cells in the tumour tissue collected from untreated animals on day 4 post-tumour inoculation consisted of Treg subsets, i.e. ~ 16.93 ± 3.88% of CD4^+^(CD8^−^)CD25^+^FOXP3^+^, known as conventional CD4^+^ Tregs, ~ 14.23 ± 3.83% of CD8^+^(CD4^−^)CTLA-4^+^ Tregs and that these proportions increased rapidly along with tumour progression to 26.06 ± 3.37% and 35.85 ± 3.53%, respectively, on day 11 (Fig. 6A). This points to an interesting observation that CD8^+^ Tregs infiltrate this tumour model more aggressively than the CD4^+^ Treg counterparts, and gives wider implications, since CD8^+^ Tregs have been previously demonstrated to have more potent tumour suppressive functions than CD4^+^ Tregs (Churlaud et al. 2015; Dai et al.^2014^). Strikingly, the SDT treatment described here appears to disrupt this pattern, since the results demonstrate a significant decrease in percentages of CD4^+^(CD8^−^)CD25^+^FOXP3^+^ and CD4^+^(CD8^−^)CD25^+^FOXP3^+^CTLA-4^+^ Tregs in tumours isolated from mice treated with the SDT compared to the untreated, as soon as day 4 after treatment (Fig. 6A), with an effect size for both populations considered as large (Supplementary Table S2). On day 11, statistically significant reduction in the proportions of the same Treg subsets was still seen with the same large magnitude of effect, accompanied by a significant decline in CD8^+^(CD4^−^) CTLA-4^+^ Tregs, which also proved to be of large effect size (Fig. 6A; Supplementary Table S2). Furthermore, given the pivotal inhibitory role of immune checkpoint molecule CTLA-4 on antitumor T cell responses and in promoting tumour immune evasion (Weber 2008), the level of CTLA-4 expression on different Treg phenotypes in tumour tissue was compared and it was found that this molecule is significantly downregulated on CD4^+^(CD8^−^)CD25^+^FOXP3^+^ Tregs in response to SDT, on day 4 (large effect size) and even by a higher magnitude of statistical significance and effect size on day 11 (Fig. 6B; Supplementary Table S2). Additionally, a decrease in CTLA-4 expression on CD4^−^CD8^−^ subset was observed, which was statistically significant and of large effect size on day 11, though not on day 4 (Fig. 6b; Supplementary Table S2).

**Fig. 7:**
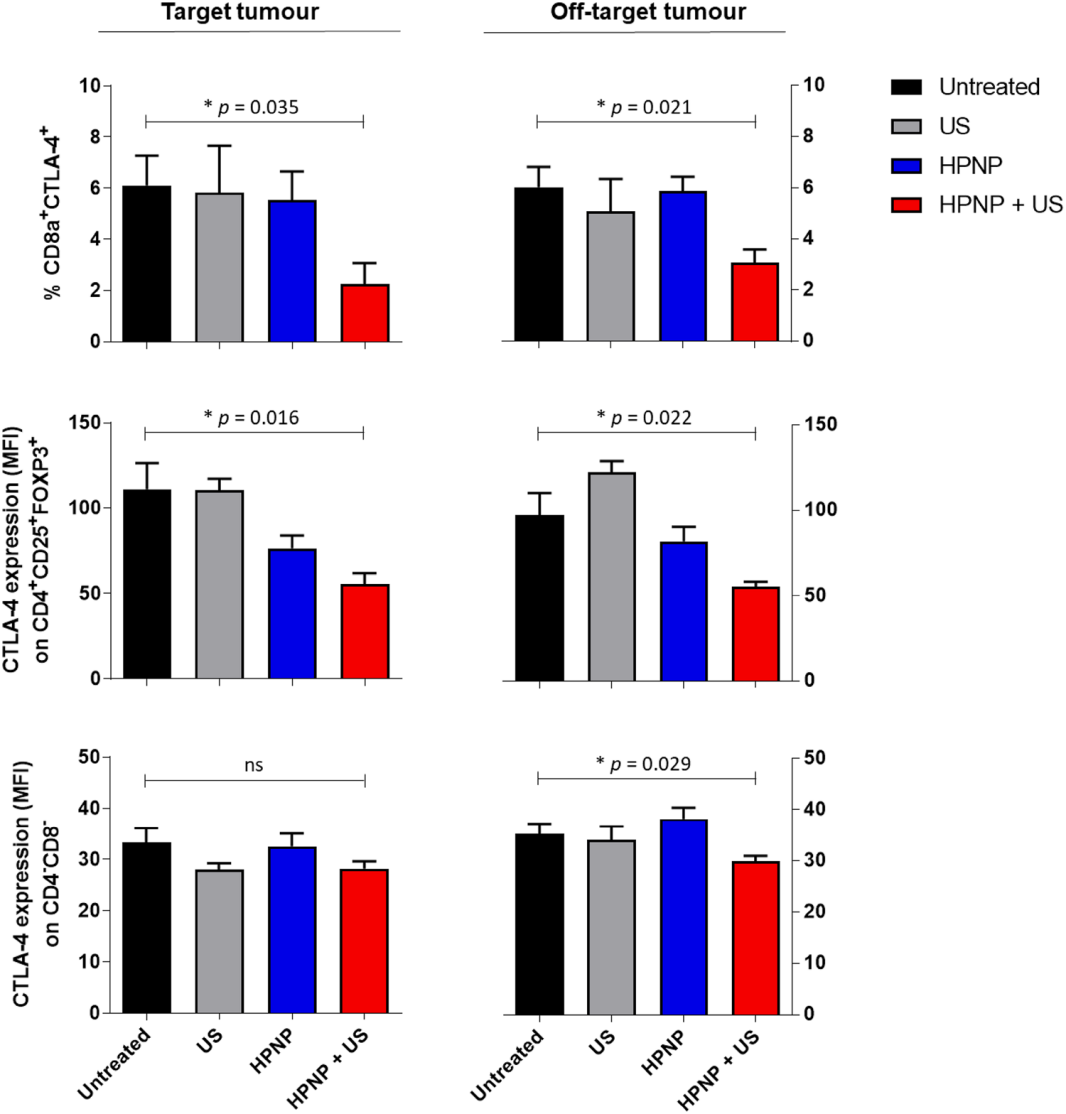
Modulation of CTLA-4-dependent suppression in primary versus distant tumour tissue in response to the SDT treatment. Primary and distant tumours were collected from mice on day 11 and cells were isolated and phenotyped by FACS analysis. Data show either the percentages of indicated CTLA-4 ^+^ cell subsets within CD45 ^+^ CD3 ^+^ T cell population or the levels of CTLA-4 expression (MFI) on given cell subsets. Statistical significance was computed using Student’s *t* test or Mann–Whitney *U* test (*ns *not significant, *p < 0.05, **p < 0.01 and ***p < 0.001). Data are expressed as mean ± SEM (*n* = 4)

In the current study, the secondary, off-target tumours that were not directly exposed to US in SDT-treated animals, represented untreated metastases in a clinical context. Encouraged by the promising results of the SDT effect on the size reduction of distant/off-target tumours, the phenotypic profile of T lymphocytes and their involvement in overriding the CTLA-4-mediated suppression in these tumours were also investigated and compared with the corresponding aspects in target tumours. Data in Fig. 7 demonstrate that SDT can significantly diminish CD8^+^(CD4^−^)CTLA-4^+^ Treg population, not only in primary tumours, in which it accounts for almost a three-fold decrease in percentage, but also by a two-fold decrease in distant tumours. In both cases the magnitude of effect size was estimated as large (Fig. 7; Supplementary Table S2). Moreover, CTLA-4 expression on conventional CD4^+^(CD8^−^)CD25^+^FOXP3^+^ Tregs was substantially downregulated by SDT (Fig. 7; Supplementary Table S2) – by two-fold in both target and off-target tumours (large effect size for both). Finally, the CTLA-4 expression on CD4^−^CD8^−^ subset was also found to be reduced in both (Fig. 7; Supplementary Table S2). However, only in off-target tumours this effect was statistically significant, and the effect size considered as large. Overall, a single SDT treatment in this study invoked a consistent decline across all scrutinised immunosuppressive parameters by a cumulative average of ~ 40%, with consistent large magnitude of effect in addition to statistical significance. This suggests high probability of a meaningful biological impact that this treatment approach is likely to have in terms of immune mechanisms. Taking into consideration the arrest of tumour progression seen in the current animal model, it appears to effectively contribute to tumour rejection.

## Discussion

For the first-line treatment of inoperable or borderline resectable PDAC with chemotherapy or radiotherapy, the low-vascularised and poorly diffused tumour microenvironment pose significant challenge (Tao et al. 2021). The efficiency of conventional treatments to eliminate/control the disease are undoubtedly hampered by the poor diffusion of agents throughout the diseased site and the low oxygen levels. The HPNP formulation employed in this study has the attribute of being degraded in the tumour microenvironment by cathepsin B, expressed and secreted by the PDAC cell lines tested here, and forms smaller particles that can, in theory, diffuse more efficiently throughout a tumour mass upon extravasation to reach poorly vascularized areas, and improve the efficacy of SDT. The levels of extracellular, i.e. secreted, cathepsin B are particularly important for nanoparticle digestion in the extracellular environment. Importantly, despite their suggested pro-survival phenotype that is associated with poor response to treatments, PANC-1 and the KPC mouse-derived cells demonstrated a 10-fold and 20-fold increase in cathepsin B secretion and activity levels (Supplementary Fig. S2), respectively, compared to that produced by BxPC-3 cells. This observation can be explained by the mutated *Kras* of the former cell types, which contributes to the altered distribution and increased secretion of cathepsin B (Cavallo-Medved et al. 2003). In addition, protonation of the HPNP and its degradation fragments in the acidic tumour environment can neutralize their highly negative charge, improving interaction with cancer cells and subsequent cellular uptake, as described by Hadi et al. (2021). Interestingly, both HPNP and the building block of the nanoparticles, poly(l-glutamic acid-l-tyrosine) 4:1, impaired cell invasion and this is an aspect that requires further investigation to uncover the mechanism that underlies this result.

Importantly, both human PDAC cell lines tested here responded well to a single SDT treatment, even at hypoxic conditions, where ROS generation may be expected to be less efficient. Any relative resistance to SDT at normoxic conditions and physiological pH observed in this study (Fig. 2B), particularly for BxPC-3 cells, is expected to be counterbalanced by the increased HPNP delivery to those more accessible parts of the tumour in an in vivo setting, since these conditions represent the adequately vascularised periphery of PDAC tumours (Zhang et al. 2018). Importantly, SDT using the HPNP formulation impaired the ability of both BxPC-3 and PANC-1 cells to form colonies and the effect was stronger against the former cell line. As demonstrated in previous studies, the PANC-1 cell line is particularly resistant to most treatment modalities, a finding that has been attributed to its mesenchymal nature and the observation that it harbours a *Kras*^G12D^ mutation which is associated with elevated pro-survival autophagy (Guo and Zhao 2021). BxPC-3, on the other hand, is a KRAS wild-type cell line and shows a greater response to treatment, including SDT.

The timing for US exposure of the target tumour is of major importance for potentiating the sonodynamic effect, not only in terms of ROS-induced tissue ablation, but also in terms of the induction of antitumour immune response and the potential to successfully apply repeated treatments (Gao et al. 2013). Nanoparticles, such as the HPNP formulation, can circulate in the vasculature system for a while after systemic administration, and exposure to US during that time can damage blood vessels that could supply the target tumour with more HPNP in subsequent repeated SDT treatments, which may be necessary to eliminate the disease. Blood vessel damage can also obstruct the infiltration of immune cells, while damage of the already limited functional tumour lymphatic vessels can impair tumour antigen presentation in the lymph nodes for the induction of antitumour immune response, as discussed later in this Section. Here, the HPNP biodistribution and tumour accumulation kinetics study informed the identification of a time point (24 h), associated with adequate tumour accumulation and minimum blood circulation of HPNP, for US exposure of the target lesion, in order to achieve a sonodynamic effect at its maximum.

The immune-related mechanisms of SDT remain yet to be fully unravelled, although gradual advances are being made. The current study is focused on the analysis of regulatory T cell subsets and the CTLA-4 immune checkpoint, known to be responsible, at least in part, for tumour immune evasion (Weber 2008). It was discovered that the SDT system employed in this study curbs a repertoire of Treg populations and leads to a reduction in CTLA-4 receptor levels. Given that these factors are among the most fundamental and persistent hallmarks of an immune-privileged tumour microenvironment that can greatly thwart efficacy of cancer therapies (Weber 2008), the proven capacity of the proposed SDT treatment to counteract these critical tumour immune escape mechanisms strongly demonstrates its high therapeutic potential. Interestingly, it has been found that tumours formed with KPC mouse-derived cells have a consistently increased abundance of immunosuppressive tumour-associated macrophages and a relatively low level of effector T cell infiltration, which is an immunological profile that characterizes human PDAC tumours (Pham et al. 2021; Blando et al. 2019). It has also been experimentally shown that the exclusion of effector T cells by KPC-derived tumours is independent of the site of tumour induction. Immune evasion in KPC tumours can be attributed to insufficient T cell priming and the lack of strong immunogenic antigens. Importantly, a single SDT treatment described here limited the immune-supressing hallmarks of this tumour model and led to cancer arrest as a standalone system, without supplementation with any additional enhancers, such as immune-checkpoint inhibitors. However, Nesbitt et al. achieved a similar effect for corresponding target and off-target tumours with an SDT platform using sensitizer-carrying microbubbles and multiple treatments within an 11-day period, in the presence of an immune checkpoint PD-L1 inhibitor (Nesbitt et al. 2021). Moreover, in a study by Yue et al., the SDT alone, in the absence of an immune checkpoint PD-1 inhibitor and an immune adjuvant (the toll-like receptor-7 agonist, imiquimod) in their nanoparticulate formulations, failed to affect distant tumours, whilst suppressing the primary. Only in combination with anti-PD-L1 blockade their SDT system managed to control both target and off-target tumours (Yue et al. 2019). Importantly and in the context of the treatment described in the current study, it is important to consider/acknowledge the previously demonstrated immunomodulatory function of the potential fragments from the enzymatic degradation of the HPNP building block, poly(L-glutamic acid-L-tyrosine) 4:1. It has been found that the infection-mimicking polyglutamic acid, at 5 kDa, can be successfully used as an immunoadjuvant to effectively elicit an anti-tumour immune response in a dose-dependent manner, with stimulation of toll-like receptor 4 having been suggested as a mechanism (Seth et al. 2015). More specifically, polyglutamic acid can facilitate dendritic cell activation, maturation, antigen uptake, migration to lymph nodes, priming of lymphocytes (including cross-presentation) in order to generate a balanced adaptive immune response.

Treg subsets that appear to be impacted by the current SDT approach included CD4^+^, CD8^+^, as well as CD4^−^CD8^−^ phenotypes. Treg cells can suppress effector T-cell responses and render the antigen-presenting cells (e.g. dendritic cells) tolerogenic and anti-inflammatory, and thus unable to prime effector T cell responses. Treg-mediated suppression mechanisms involve secretion of inhibitory cytokines such as IL-10 and TGF-β, or cell contact-dependent mechanisms, such as those mediated by CTLA-4 (Ohue and Nishikawa 2019). For example, CD8^+^ Tregs isolated from prostate or colorectal tumours are commonly characterized by CTLA-4 expression and TGF-β production, suppressing CD4^+^ T cell proliferation, Th1 cell expansion and their IFN-γ production ex vivo. Overall, it is well established that tumour infiltrating CD8^+^ Tregs largely contribute to tumour immune escape and their levels correlate with cancer progression (Churlaud et al. 2015; Vieyra-Lobato et al. 2018; Yu et al. 2018). Therefore, not surprisingly, the depletion of this cell subset (among others) by the SDT-based treatment must have contributed to the cancer growth inhibition.

The immune checkpoint-mediated immunosuppression specifically may play a vital role in metastasis and consequently, blocking them with anti-PD-L1/PD-1 or anti-CTLA-4, could offer an important therapeutic advantage. The use of checkpoint inhibitors to cease regulatory mechanisms and their potential for cancer immunotherapy is now well-recognised. The therapeutic effect of anti-CTLA-4 antibodies in cancer can be attributed to selective depletion of tumour-infiltrating Tregs (Liu and Zheng 2020). Therefore, an inherent capacity of SDT to release these Treg and CTLA-4 breaks on antitumour immunity offers unique benefits for a cancer therapy. Even more so considering that clinically tested anti-CTLA-4 antibodies are characterised by suboptimal efficacy and high toxicity (Liu and Zheng 2020).

The occurrence of metastatic spread is undoubtedly associated with poor prognosis, particularly in pancreatic cancer, as curative surgical resection is not an option (Du et al. 2016; Giovannetti et al. 2017). The results in Fig. 5 depict that SDT treatment had a remarkable effect against off-target tumors, which were not exposed to US. This phenomenon, termed as abscopal effect, occurs when a type of local therapy, not only shrinks the target tumour but also leads to the shrinkage of untreated distant tumours. This effect has been previously observed in both preclinical studies and, rarely, in clinical studies, in patients with metastatic progression that have been treated with sonodynamic therapy, radiotherapy, including proton beam therapy, cryoablation and radiofrequency ablation (Wang et al. 2009; Inui et al. 2014). The abscopal effect of SDT was previously explained by Zhang, et al. (Zhang et al. 2018), who, using preclinical animal models, discovered that the debris from tumour cells destroyed by SDT could serve as a source of tumour antigens to stimulate host antitumour immune responses. This hypothesis has also been confirmed in the context of preliminary clinical trial studies in breast cancer (Wang et al. 2009; Inui et al. 2014). SDT is a non-hyperthermic ablation approach, which, unlike many of the alternative approaches, preserves the structural integrity of any tumour neoantigens that are generated during or following treatment. Upon primary tumour damage, tumour-associated antigens become exposed to and are taken up by dendritic cells, which then present them to T cells in lymph nodes and orchestrate systemic T cell responses. However, tumour-infiltrating Tregs present in the tumour microenvironment, especially those expressing CTLA-4, intercept dendritic cells and render them tolerogenic and anti-inflammatory, which eventually stimulates T cell exhaustion instead of activation (Facciabene et al. 2012; Gardner and Ruffell 2016; Ohue and Nishikawa 2019). Since SDT appears to hold CTLA-4-mediated suppression and Tregs at bay, it likely permits more dendritic cells to adopt mature immunocompetent phenotype and thus more effectively induce effector T cell responses. This should consequently translate to an improved systemic antitumour immunity and tumour rejection.

## Conclusions

In these studies, HPNP-mediated SDT was used to treat a number of pancreatic cancer cell lines in vitro and ectopic pancreatic tumours in immunocompetent mice. Examining the effect of different combination of US parameters with positive sonodynamic effect for a specific treatment duration, it was found that the overall acoustic energy provided per unit area and not the US intensity affects the outcome of the treatment. This minimally invasive therapeutic modality also appeared to disrupt immunosuppressive mechanisms in the tumour microenvironment, which likely restored antitumor immunity and accordingly halted the primary and distant tumour progression. Overall, the findings from the current study present compelling evidence that SDT using the HPNP formulation has significant potential as a monotherapy or adjunctive treatment of unresectable or border-line resectable pancreatic cancer, aiming to either downstage or progressively, fully eliminate the disease.

## Supplementary Information

Below is the link to the electronic supplementary material.

Supplementary file1 (DOCX 127 KB)

## Data Availability

The datasets used and/or analyzed during the current study are available from the corresponding author on reasonable request.

## Abbreviations

CTLA-4: Cytotoxic T-lymphocyte-associated protein 4
DC: Duty cycle
DMSO: Dimethyl sulfoxide
EDTA: Ethylenediamine tetraacetic acid
FBS: Foetal bovine serum
FOXP3: Forkhead box P3
HP: Hematoporphyrin
HPNP: Hematoporphyrin-carrying nanoparticles formed with the poly(l-glutamic acid-l-tyrosine co-polymer
IFN-γ: Interferon γ
MTT: 3-(4,5-Dimethylthiazol-2-yl)-2,5-diphenyl-2H-tetrazolium bromide
PBS: Phosphate buffered saline
PD-1: Programmed cell death protein 1
PDAC: Pancreatic ductal adenocarcinoma
PD-L1: Programmed death-ligand 1
ROS: Reactive oxygen species
SDT: Sonodynamic therapy
SFM: Serum-free medium
TGF-β: Transforming growth factor β
Th1: T helper 1
Tregs: Regulatory T cells
US: Ultrasound

## References

[CR1] Blando J, Sharma A, Higa MG (2019) Comparison of immune infiltrates in melanoma and pancreatic cancer highlights vista as a potential target in pancreatic cancer. Proc Natl Acad Sci USA 116:1692–1697 30635425 10.1073/pnas.1811067116 PMC6358697

[CR2] Cavallo-Medved D, Dosescu J, Linebaugh BE, Sameni M, Rudy D, Sloane BF (2003) Mutant K-ras regulates cathepsin B localization on the surface of human colorectal carcinoma cells. Neoplasia 5:507–519 14965444 10.1016/s1476-5586(03)80035-0 PMC1502576

[CR3] Churlaud G, Pitoiset F, Jebbawi F, Lorenzon R, Bellier B, Rosenzwajg M, Klatzmann D (2015) Human and mouse CD8(+)CD25(+)FOXP3(+) regulatory T cells at steady state and during interleukin-2 therapy. Front Immunol 6:171 25926835 10.3389/fimmu.2015.00171 PMC4397865

[CR4] Civale J, Bamber J, Rivens I, ter Haar G (2006) Optimising HIFU lesion formation with backscatter attenuation estimation (BAE). AIP Conf Proc 829:176

[CR5] Dai Z, Zhang S, Xie Q, Wu S, Su J, Li S, Xu Y, Li XC (2014) Natural CD8^+^CD122^+^ T cells are more potent in suppression of allograft rejection than CD4^+^CD25^+^ regulatory T cells. Am J Transpl 14:39–48 10.1111/ajt.12515 24219162

[CR6] Das M, Shen L, Liu Q, Goodwin TJ, Huang L (2019) Nanoparticle delivery of RIG-I agonist enables effective and safe adjuvant therapy in pancreatic cancer. Mol Ther 27:507–517 30545600 10.1016/j.ymthe.2018.11.012 PMC6401191

[CR7] Du YX, Liu ZW, You L, Wu WM, Zhao YP (2016) Advances in understanding the molecular mechanism of pancreatic cancer metastasis. Hepatobil Pancreat Dis Int 15:361–370 10.1016/s1499-3872(15)60033-9 27498575

[CR8] Facciabene A, Motz GT, Coukos G (2012) T-regulatory cells: key players in tumor immune escape and angiogenesis. Cancer Res 72:2162–2171 22549946 10.1158/0008-5472.CAN-11-3687 PMC3342842

[CR9] Gao Z, Zheng J, Yang B (2013) Sonodynamic therapy inhibits angiogenesis and tumor growth in a xenograft mouse model. Cancer Lett 335:93–99 23402818 10.1016/j.canlet.2013.02.006

[CR10] Gardner A, Ruffell B (2016) Dendritic cells and cancer immunity. Trends Immunol 37:855–865 27793569 10.1016/j.it.2016.09.006 PMC5135568

[CR11] Giovannetti E, van der Borden CL, Frampton AE, Ali A, Firuzi O, Peters GJ (2017) Never let it go: stopping key mechanisms underlying metastasis to fight pancreatic cancer. Semin Cancer Biol 44:43–59 28438662 10.1016/j.semcancer.2017.04.006

[CR12] Göbl R, Virga S, Rackerseder J, Frisch B, Navab N, Hennersperger C (2017) Acoustic window planning for ultrasound acquisition. Int J Comput Assist Radiol Surg 12:993–1001 28285339 10.1007/s11548-017-1551-3 PMC5447334

[CR13] Guillaumond F, Leca J, Olivares O, Lavaut MN, Vidal N, Berthezene P, Dusetti NJ, Loncle C, Calvo E, Turrini O, Iovanna JL, Tomasini R, Vasseur S (2013) Strengthened glycolysis under hypoxia supports tumor symbiosis and hexosamine biosynthesis in pancreatic adenocarcinoma. PNAS 110:3919–3924 23407165 10.1073/pnas.1219555110 PMC3593894

[CR14] Guo C, Zhao Y (2021) Autophagy in pancreatic cancer. J Mol Cell Biol 13:786–790 10.1093/jmcb/mjab053 PMC8782586 34459894

[CR15] Hadi MM, Nesbitt H, Masood H, Sciscione F, Patel S, Ramesh BS, Emberton M, Callan JF, MacRobert A, McHale AP, Nomikou N (2021) Investigating the performance of a novel pH and cathepsin B sensitive, stimulus-responsive nanoparticle for optimised sonodynamic therapy in prostate cancer. J Control Rel 329:76–86 10.1016/j.jconrel.2020.11.040 PMC8551370 33245955

[CR16] Inui T, Makita K, Miura H, Matsuda A, Kuchiike D, Kubo K, Mette M, Uto Y, Nishikata T, Hori H, Sakamoto N (2014) Case report: a breast cancer patient treated with GcMAF, sonodynamic therapy and hormone therapy. Anticancer Res 34:4589–4593 25075104

[CR17] Lakens D (2013) Calculating and reporting effect sizes to facilitate cumulative science: a practical primer for t-tests and ANOVAs. Front Psychol 4:863 24324449 10.3389/fpsyg.2013.00863 PMC3840331

[CR18] Liu Y, Zheng P (2020) Preserving the CTLA-4 checkpoint for safer and more effective cancer immunotherapy. Trends Pharmacol Sci 41:4–12 31836191 10.1016/j.tips.2019.11.003 PMC7210725

[CR19] McHale AP, Callan JF, Nomikou N, Fowley C, Callan B (2016) Sonodynamic therapy: concept, mechanism and application to cancer treatment. Adv Exp Med Biol 880:429–450 26486350 10.1007/978-3-319-22536-4_22

[CR20] Nesbitt H, Logan K, Thomas K, Callan B, Gao J, McKaig T, Taylor M, Love M, Stride E, McHale AP, Callan JF (2021) Sonodynamic therapy complements PD-L1 immune checkpoint inhibition in a murine model of pancreatic cancer. Cancer Lett 517:88–95 34119606 10.1016/j.canlet.2021.06.003

[CR21] Nicholas D, Nesbitt H, Farrell S, Logan K, McMullin E, Gillan T, Kelly P, O’Rourke D, Porter S, Thomas K, O’Hagan BMG, Nomikou N, Callan B, Callan JF, McHale AP (2021) Exploiting a rose bengal-bearing, oxygen-producing nanoparticle for SDT and associated immune-mediated therapeutic effects in the treatment of pancreatic cancer. Eur J Pharm Biopharm 163:49–59 33798727 10.1016/j.ejpb.2021.03.005

[CR22] Niknafs N, Zhong Y, Moral JA, Zhang L, Shao MX, Lo A, Makohon-Moore A, Lacobuzio-Donahue CA, Karchin R (2019) Characterization of genetic subclonal evolution in pancreatic cancer mouse models. Nat Commun 10:5435 31780749 10.1038/s41467-019-13100-w PMC6882784

[CR23] Nomikou N, Curtis K, McEwan C, O’Hagan BMG, Callan B, Callan JF, McHale AP (2017) A versatile, stimulus-responsive nanoparticle-based platform for use in both sonodynamic and photodynamic cancer therapy. Acta Biomater 49:414–421 27856283 10.1016/j.actbio.2016.11.031

[CR24] Ohue Y, Nishikawa H (2019) Regulatory T (Treg) cells in cancer: can Treg cells be a new therapeutic target? Cancer Sci 110:2080–2089 31102428 10.1111/cas.14069 PMC6609813

[CR25] Peng Y, Jia L, Wang S, Cao W, Zheng J (2018) Sonodynamic therapy improves antitumor immune effect by increasing the infiltration of CD8^+^ T cells and altering tumor blood vessels in murine B16F10 melanoma xenograft. Oncol Rep 40:2163–2170 30106435 10.3892/or.2018.6612

[CR26] Pham TND, Shields MA, Spaulding C, Principe DR, Li B, Underwood PW, Trevino JG, Bentrem DJ, Munshi HG (2021) Preclinical models of pancreatic ductal adenocarcinoma and their utility in immunotherapy studies. Cancers 13:440 33503832 10.3390/cancers13030440 PMC7865443

[CR27] Seth A, Heo MB, Sung MH, Lim YT (2015) Infection-mimicking poly(γ-glutamic acid) as adjuvant material for effective anti-tumor immune response. Int J Biol Macromol 75:495–504 25709015 10.1016/j.ijbiomac.2015.02.013

[CR28] Soltani M, Souri M, Kashkooli MF (2021) Effects of hypoxia and nanocarrier size on pH-responsive nano-delivery system to solid tumors. Sci Rep 11:19350 34588504 10.1038/s41598-021-98638-w PMC8481507

[CR29] Tao J, Yang G, Zhou W, Qui J, Chen G, Luo W, Zhao F, You L, Zheng L, Zhang T, Zhao Y (2021) Targeting hypoxic tumor microenvironment in pancreatic cancer. J Hematol Oncol 14:14 33436044 10.1186/s13045-020-01030-w PMC7805044

[CR30] Thomas RG, Surendran SP, Jeong YY (2020) Tumor microenvironment-stimuli responsive nanoparticles for anticancer therapy. Front Mol Biosci 7:610533 33392264 10.3389/fmolb.2020.610533 PMC7775573

[CR31] Vieyra-Lobato MR, Vela-Ojeda J, Montiel-Cervantes L, Lopez-Santiago R, Moreno-Lafont MC (2018) Description of CD8(^+^) regulatory T lymphocytes and their specific intervention in graft-versus-host and infectious diseases, autoimmunity, and cancer. J Immunol Res 2018:3758713 30155493 10.1155/2018/3758713 PMC6098849

[CR32] Wang X, Zhang W, Xu Z, Luo Y, Mitchell D, Moss RW (2009) Sonodynamic and photodynamic therapy in advanced breast carcinoma: a report of 3 cases. Integr Cancer Ther 8:283–287 19815599 10.1177/1534735409343693

[CR33] Weber JS (2008) Tumor evasion may occur via expression of regulatory molecules: a case for CTLA-4 in melanoma. J Invest Dermatol 128:2750–2752 18997841 10.1038/jid.2008.341

[CR34] Yu Y, Ma X, Gong R, Zhu J, Wei L, Yao J (2018) Recent advances in CD8(^+^) regulatory T cell research. Oncol Lett 15:8187–8194 29805553 10.3892/ol.2018.8378 PMC5950136

[CR35] Yue W, Chen L, Yu L, Zhou B, Yin H, Ren W, Liu C, Guo L, Zhang Y, Sun L, Zhang K, Huixiong X, Chen Y (2019) Checkpoint blockade and nanosonosensitizer-augmented noninvasive sonodynamic therapy combination reduces tumour growth and metastases in mice. Nat Commun 10:2025 31048681 10.1038/s41467-019-09760-3 PMC6497709

[CR36] Zhang Z, Ji S, Zhang B, Liu J, Qin Y, Xu J, Yu X (2018) Role of angiogenesis in pancreatic cancer biology and therapy. Biomed Pharmacother 108:1135–1140 30372814 10.1016/j.biopha.2018.09.136

[CR37] Zhang Q, Bao C, Cai X, Jin L, Sun L, Lang Y, Li L (2018) Sonodynamic therapy-assisted immunotherapy: A novel modality for cancer treatment. Cancer Sci 109:1330–1345 29575297 10.1111/cas.13578 PMC5980136

