# Correction to: Nanotechnology-augmented sonodynamic therapy and associated immune-mediated effects for the treatment of pancreatic ductal adenocarcinoma

**DOI:** 10.1007/s00432-023-04648-8

**Published:** 2023-03-22

**Authors:** Marym Mohammad Hadi, Sian Farrell, Heather Nesbitt, Keith Thomas, Ilona Kubajewska, Alex Ng, Hamzah Masood, Shiv Patel, Fabiola Sciscione, Brian Davidson, John F. Callan, Alexander J. MacRobert, Anthony P. McHale, Nikolitsa Nomikou

**Affiliations:** 1 Division of Surgery and Interventional Science, Faculty of Medical Sciences, University College London, London, UK; 2 Biomedical Sciences Research Institute, Ulster University, Coleraine, UK; 3 Nanomerics Ltd, London, UK

**Correction to: Journal of Cancer Research and Clinical Oncology** 10.1007/s00432-022-04418-y

In Fig. 5 of this article, there were two errors in Fig. 5c, right panel. Firstly, the title of the graph (in the right panel) that had read “Target tumour” should have read “Off-target tumour”. In addition, the graph had presented the raw tumour volume data, and it should have presented the % tumour volume increase data. Asterisks that depict the level of significance had also been missing;

The Fig. 5c should have appeared as shown below:

**Fig. 5:**
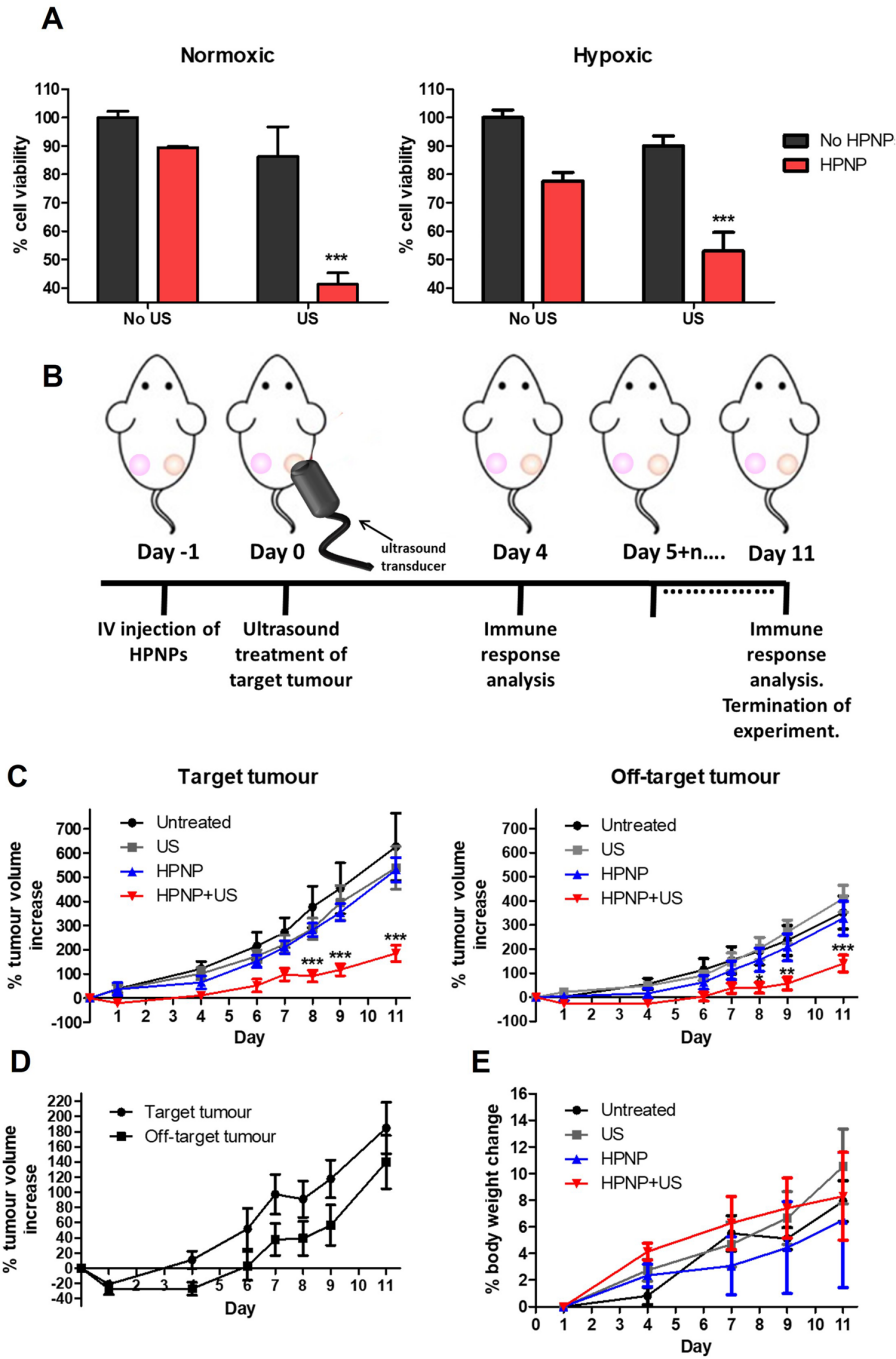
Sonodynamic treatment of T110299 tumours. **A** % cell viability of T110299 cells treated in the absence (no HPNP) and the presence of nanoparticles (HPNP), without (no US) and with ultrasound exposure at 3 W/cm^2^ and 50% DC, for 30 s, at normoxic and hypoxic conditions. **B** In vivo treatment protocol. **C** Plot of % change of target and off-target tumour volumes treated with no treatment (untreated), ultrasound only (US), nanoparticles carrying hematoporphyrin (HPNP) and nanoparticles carrying hematoporphyrin with ultrasound, i.e. sonodynamic therapy, SDT, (HPNPs + US). **D** Plot of % change of target and off-target tumour volumes trated with SDT. **E** The corresponding animal body weight increase. Statistical significance was computed using Two-way ANOVA with Bonferroni post-test (**A**) and One-way ANOVA with Tukey multiple comparison test (**C**) (*p < 0.05, **p < 0.01, ***p < 0.001). For **A**: the asterisks show the significance of difference between samples incubated in the presence and the absence of nanoparticles, under ultrasound exposure. Error bars represent ± the SD, where *n* = 5

