# Supplementary material for: Nanotechnology-augmented sonodynamic therapy and associated immune-mediated effects for the treatment of pancreatic ductal adenocarcinoma
Source: J Cancer Res Clin Oncol. 2022 Nov 2;149(8):5007–23. doi: 10.1007/s00432-022-04418-y (PMC10349707; doi:10.1007/s00432-022-04418-y)
Supplement: Supplementary file 1 — Supplementary file1 (DOCX 127 KB) [file 432_2022_4418_MOESM1_ESM.docx]

**Nanotechnology-augmented sonodynamic therapy and associated immune-mediated effects for the treatment of pancreatic ductal adenocarcinoma**

Marym Mohammad Hadi^1*^, Sian Farrell^2*^, Heather Nesbitt^2^, Keith Thomas^2^, Ilona Kubajewska^1,3^, Alex Ng^1^, Hamzah Masood^1^, Shiv Patel^1^, Fabiola Sciscione^1^, Brian Davidson^1^, John F. Callan^2^, Alexander J. MacRobert^1^, Anthony P. McHale^2^, Nikolitsa Nomikou^1†^

1. Division of Surgery & Interventional Science, Faculty of Medical Sciences, University College London, UK

2. Biomedical Sciences Research Institute, Ulster University, Coleraine, UK

3. Nanomerics Ltd., London, UK

* Joint first authors

† Corresponding author

**Supplementary Material**

**1. “Silent” dose response to HP and HPNP**

**Method**

BxPC-3, PANC-1 and T110299 cells were seeded in 96-well plates at a concentration of 2 × 10^4^, 2 x 10^4^, and 1× 10^4^ cells per well, respectively. The systems were incubated, in a humidified 5% CO_2_/20% O_2_ atmosphere, at 37°C, for 24 h. The growth medium was then replaced with fresh medium containing free HP or HPNP, at concentrations ranging from 0.01 to 20 μg/mL based on HP, at either pH 6.4 or pH 7.4. The control systems contained only growth medium at the corresponding pH, in the absence of a sensitizing agent. Cells were incubated for 24 h and treatment medium was then removed, cell monolayers were washed with PBS and fresh growth medium was added in each well. The plates were then incubated for 24 h and cell viability was determined using an MTT assay.

**Results**

The manufacturing, physicochemical characteristics and performance of the stimulus-responsive nanoparticulate platform (HPNP) used in the current study for cancer sonodynamic therapy (SDT) has been previously described. In order to examine the synergistic effect of the nanoparticles in combination of ultrasound, which constitutes the principle of SDT, it was important to identify the maximum concentration of nanoparticulate hematoporphyrin with a relative low cytotoxicity against the target human pancreatic cell lines, BxPC-3 and PANC-1, in the absence of ultrasound (“silent” toxicity). The toxicity profile of the nanoparticles has previously been found to be affected by the pH conditions. Hence, in this study, it was essential to determine the toxicity profile of the nanoparticles and compare it with that of free hematoporphyrin, at pH 7.4 and 6.4, the former pH representing the well-oxygenated healthy tissues and well-vascularized tumour borders, and the latter representing the hypoxic inner tumour microenvironment. BxPC-3 and PANC-1 cells were treated with increasing concentrations of the free HP or HPNPs, ranging from 0.01-20 µg/mL based on HP concentration. Results in Fig. S1 demonstrate that, for BxPC-3 cells, the free and nanoparticulate HP significantly reduced cell viability at concentrations higher than 10 µg/mL at both pH 7.4 and 6.4, while the free sensitizer did not have any cytotoxic effect at concentrations up to 20 µg/mL. The “silent” toxicity profile of PANC-1 and T110299 cells showed that the highest concentration of HPNP with relatively low cytotoxic effect was 15 µg/mL, for both pH conditions.

**Figure S1**


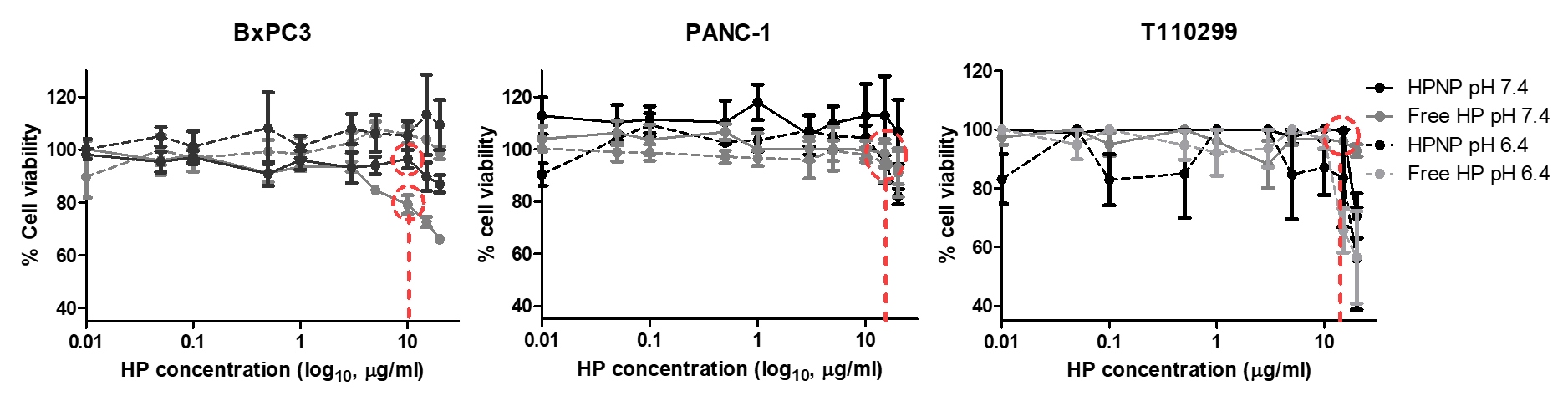


**“Silent” dose response to free HP and HPNP.** Cytotoxicity of free HP and HPNP , at pH 7.4 and pH 6.4, for BxPC-3, PANC-1 and T110299 cells. The concentrations used in following experiments for pH 6.4 and pH 7.4, for each cell line, are indicated in red (n=3).

**2. Cathepsin B assay**

**Figure S2**


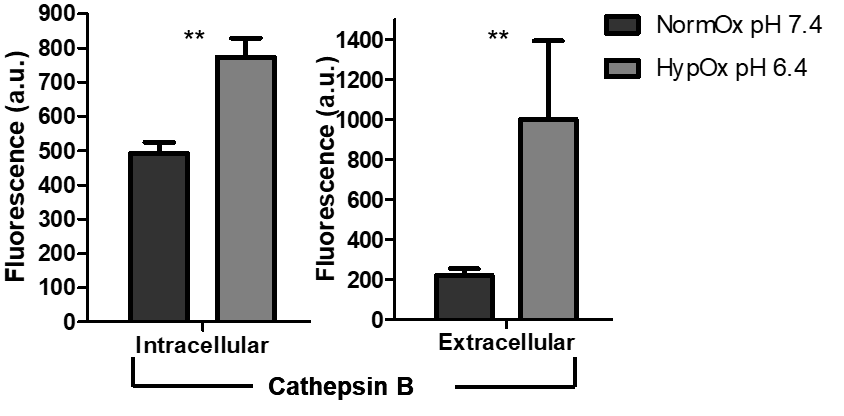


**Cathespin B assay:** Levels of extracellular and intracellular cathepsin B after 24 h incubation of T110299 cells at normoxic condition and pH 7.4 (NormOx 7.4) and at hypoxic conditions and pH 6.4 (HypOx 6.4).

**3.** **poly(L-glutamic acid-L-tyrosine) 4:1 cell toxicity study**

**Method**

Cells were seeded in 96 well-plates at a density of 2 × 10^4^ cells per well and were incubated at 37°C, in a 5% CO2 humidified atmosphere, for 24 h. Each cell line was incubated at either pH 7.4 or pH 6.4 in the presence of poly(L-glutamic acid-L-tyrosine) 4:1 (Merck, UK) at concentrations ranging from 0.120 – 1.240 mg/mL. The control systems contained only growth medium at the corresponding pH, in the absence of the co-polymer. The plates were incubated under normoxic conditions in a 5% CO_2_ humidified atmosphere for 24 h. The treatment solutions were then removed, cells were washed with PBS and fresh growth medium was added. The plates were further incubated in normoxic conditions for 24 h. Cell viability was determined using an MTT assay.

**Results**

**Figure S3**


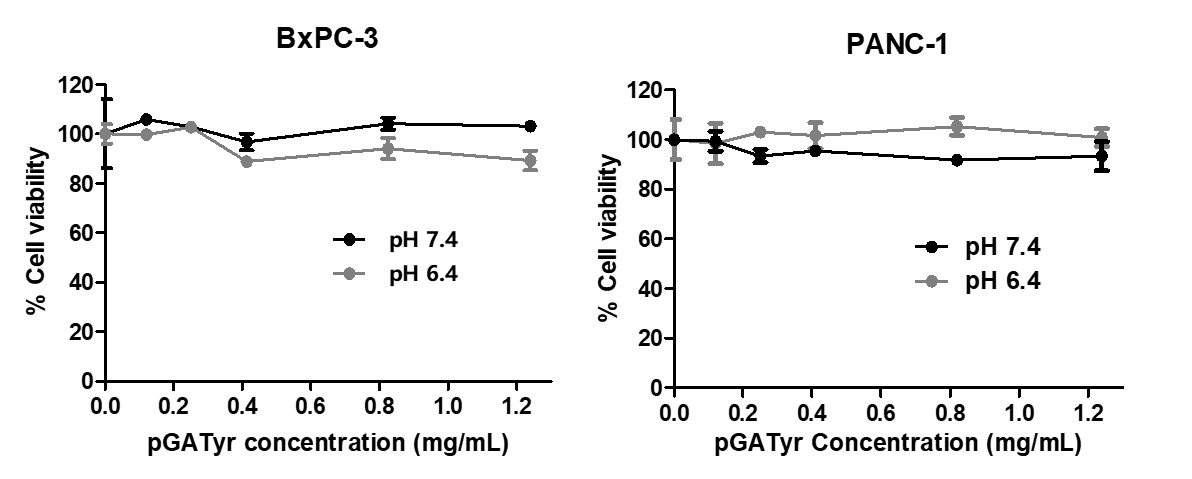


**Dose response to** **poly(L-glutamic acid-L-tyrosine) 4:1 (pGATyr)**. Cytotoxicity of pGATyr, at pH 7.4 and pH 6.4, for BxPC-3 and PANC-1 cells (n=3).

**4. In vivo SDT efficacy studies**

**Figure S4**


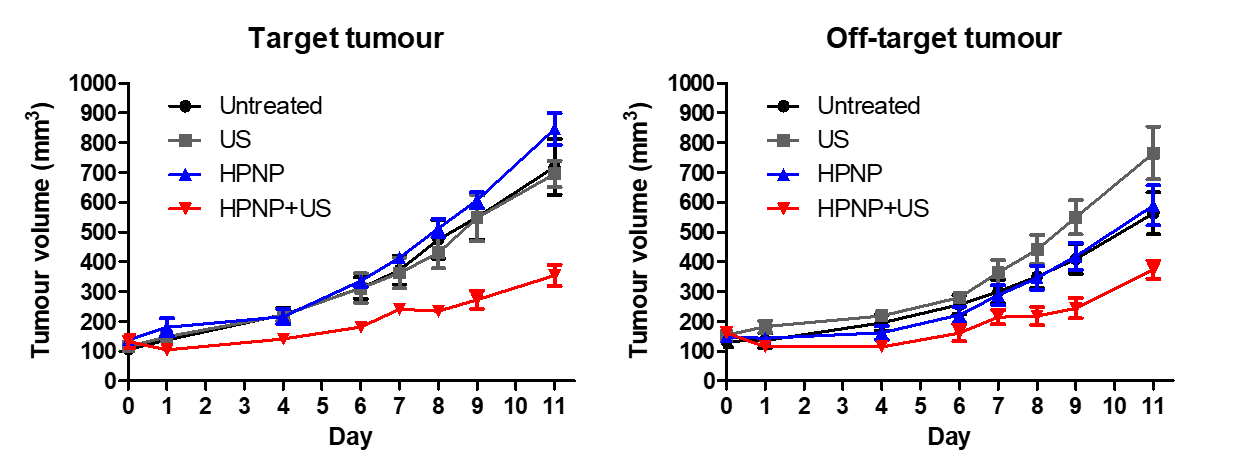


**Sonodynamic treatment of T110299 tumours.** Plot for the volumes of target and off-target tumours treated with no treatment (Untreated), ultrasound only (US), nanoparticles carrying hematoporphyrin (HPNP) and nanoparticles carrying hematoporphyrin with ultrasound, i.e. SDT (HPNP+US).

**5. Immune mechanisms of SDT antitumour activity**

**Table S1.** Flow cytometry gating strategy.

| PBMCs | CD45 | CD3 | CD4 | CD8a | CD25 | FoxP3 |
| --- | --- | --- | --- | --- | --- | --- |
| T Helper | + | + | + | - | - | - |
| Cytotoxic | + | + | - | + | - | - |
| T _Reg_ | + | + | + | - | + | + |

**Table S2.** Effect size and statistical significance of changes in immune cell phenotypes following SDT.

| **Cell phenotype (within CD3^+^CD45^+^)** | **Collec-tion day** | **Tumour tissue** | **Group mean 1 ± SEM (Un-treated)** | **Group mean 2 ± SEM (SDT)** | **Sample size (n)** | **P value** | **Test of effect size between means** | | | **Effect size magni-tude** |
| --- | --- | --- | --- | --- | --- | --- | --- | --- | --- | --- |
|  |  |  |  |  |  |  | **Cohen’s d** | **Glass's delta** | **Hedges' g** |  |
| **% CD4^+^CD8^-^CD25^+^FOXP3^+^** | D4 | Total | 16.93 ±  3.878 | 7.688 ±  1.223 | 8 | 0.039 | 1.136297 | 0.842479 | 1.136297 | large |
|  | D10 | Total | 26.06 ±  3.367 | 17.97 ±  1.449 | 8 | 0.045 | 1.103606 | 0.849522 | 1.103606 | large |
| **% CD4^+^CD8^-^CD25^+^FOXP3^+^CTLA-4^+^** | D4 | Total | 16.93 ±  3.878 | 7.681 ±  1.222 | 8 | 0.039 | 1.137247 | 0.843118 | 1.137247 | large |
|  | D10 | Total | 26.03 ±  3.356 | 17.95 ±  1.449 | 8 | 0.044 | 1.105192 | 0.851243 | 1.105192 | large |
| **% CD8a^+^CTLA4^+^** | D10 | Total | 35.85 ±  3.534 | 27.32 ±  2.315 | 8 | 0.0499 | 1.009544 | 0.853341 | 1.009544 | large |
| **CTLA4 MFI on CD4^+^CD8^-^CD25^+^FOXP3^+^** | D4 | Total | 153.6 ±  13.73 | 106.7 ±  10.79 | 8 | 0.0178 | 1.34312 | 1.207829 | 1.34312 | large |
|  | D10 | Total | 104.0 ±  9.754 | 55.40 ±  3.290 | 8 | 0.0003 | 2.360515 | 1.761508 | 2.360515 | large |
| **CTLA4 MFI on CD4^-^CD8^-^** | D10 | Total | 35.85 ±  4.627 | 27.32 ±  2.444 | 8 | 0.014 | 1.399942 | 1.119516 | 1.399942 | large |
| **% CD8a^+^CD4^-^CTLA4^+^** | D10 | Primary | 6.103  ±  1.171 | 2.258  ±  0.8077 | 4 | 0.035 | 1.9114 | 1.641759 | 1.9114 | large |
|  | D10 | Distant | 6.028  ± 0.7946 | 3.063  ±  0.5284 | 4 | 0.021 | 2.197149 | 1.865953 | 2.197149 | large |
| **CTLA4 MFI on CD4^+^CD8^-^CD25^+^FOXP3^+^** | D10 | Primary | 111.1  ±  15.43 | 55.43  ±  6.441 | 4 | 0.016 | 2.354344 | 1.803953 | 2.354344 | large |
|  | D10 | Distant | 96.93  ±  13.14 | 55.36  ±  3.003 | 4 | 0.022 | 2.180774 | 1.581811 | 2.180774 | large |
| **CTLA4 MFI on CD4^-^CD8^-^** | D10 | Distant | 35.25  ±  1.987 | 29.94  ±  1.035 | 4 | 0.029 | 1.675763 | 1.335849 | 1.675763 | large |
